# Supplementary material for: Diverse organic molecules on Mars revealed by the first SAM TMAH experiment
Source: Nat Commun. 2026 Apr 21;17:2748. doi: 10.1038/s41467-026-70656-0 (PMC13100061; doi:10.1038/s41467-026-70656-0)
Supplement: Supplementary file 1 — Supplementary Information [file 41467_2026_70656_MOESM1_ESM.pdf]

Supplementary Information

Peak Number	Confirmed Identity	Potential ID from NIST	Standards tested	Rt min offset	Detected in Mojarro et al. (2023)?	
					SAM pyro	TMAH
**1**	**trimethylbenzene**	**benzene, trimethyl-**	**benzene, trimethyl-**	**-0.6**	**Y**	**Y**
**2**	**tetramethylbenzene**	**benzene, tetramethyl-**	**benzene, tetramethyl-**	**0.3**	**Y**	**Y**
3	?	benzenediamine, trimethyl-	benzenediamine, trimethyl-	7.6	N	N
		benzenemethanol, 4-(1-methylethyl)	*nd*	nd	N	N
4	?	benzendiamine, trimethyl-	benzendiamine, trimethyl-	6.9	N	N
		ethyltetramethylcyclopentadiene	*nd*	nd	Y	Y
		methyl 2-methyl benzoate	*nd*	nd	N	N
5	dimethylsilanediol-2TBDMS*	*n/a*	*nd*	nd	*nd*	*nd*
**6**	**fluoronaphthalene**	**fluoronaphthalene**	**fluoronaphthalene**	**0 to 0.8**	**IS**	**IS**
**7**	**naphthalene**	**naphthalene**	**naphthalene**	**0 to 0.5**	**Y**	**Y**
**8**	**benzothiophene**	**benzothiophene**	**benzothiophene**	**0 to 0.3**	**Y**	**Y**
9	?	benzenamine, N,N,2-trimethyl	benzenamine, N,N,2-trimethyl	8.1	nd	Y
10	?	benzenamine, N,N,dimethyl-	benzenamine, N,N,dimethyl-	4.1	N	N
**11**	**methyl benzoate**	**methyl benzoate**	**methyl benzoate**	**-0.2**	**nd**	**Y**
12	?	anisole, 1,4-dimethyl	*nd*	nd	N	N
		phenol, 3,4,5-trimethyl	*nd*	nd	N	N
13	?	benzene, 1,2,4,5-tetramethyl	benzene, 1,2,4,5-tetramethyl	-1.3	Y	Y
		O-cymene	*nd*	nd	N	N
		benzene, 2-ethyl, 1,4-dimethyl-	*nd*	nd	N	N
14	?	benzene, 1,4-diethyl-,	*nd*	nd	N	N
		benzene, 2-ethyl, 1,4-dimethyl-,	*nd*	nd	N	N
		benzene, 4-ethyl, 1,2-dimethyl-	*nd*	nd	N	N
**15**	**dihydronaphthalene**	**dihydronaphthalene**	**dihydronaphthalene**	**0.5**	**N**	**N**
16	?	benzene 1-methoxy-4-(1-methylethyl)	*nd*	nd	nd	Y
		phenol, 2, 3, 5, 6-tetramethyl-	*nd*	nd	N	N
17	?	benzenamine, N,N,2,4-tetramethyl-	benzenamine, N,N,2,4-tetramethyl-	3	N	N
18	?	phenol, 2, 3, 5, 6-tetramethyl-	*nd*	nd	N	N
		benzenediamine, NNN-trimethyl-1,4-	NNN-trimethyl-1,4-benzenediamine	7.2	N	N
19	?	naphthalene, 1,2-dihydro-3-methyl	naphthalene, 1,2-dihydro-3-methyl	10.2	N	N
20	?	benzene, pentamethyl-	benzene, pentamethyl-	1.3	N	N
**21**	**methylnaphthalene**	**methylnaphthalene**	**methylnaphthalene**	**1.3**	**Y**	**Y**
22	?	indole, dimethyl-	indole, dimethyl-	3.7	nd	Y
23	Diphenylmethane*	diphenylmethane	diphenylmethane	1.9	Y	Y
24	?	naphthalene, 1-methoxy	*nd*	nd	nd	Y

table S-1. List of confirmed and potential molecule identities from SAM-Flight Model TMAH experiment and analytical standards analyzed with the SAM-Breadboard and flight spare columns. Not every potential identity was compared to an analytical standard. The retention time (Rt) comparison between the molecule on SAM-Flight Model and the SAM-Breadboard is reported for those standards that were analyzed. Noted as Y for yes are those molecules that were also detected in the Murchison meteorite with SAM-like neat pyrolysis or TMAH (tetramethylammonium hydroxide) thermochemolysis from (*1*). * Denotes known SAM byproducts or background.

Known SAM-internal organic molecules. Several known SAM-internal molecules were identified in the EGA and GC data, including mono- and bisilylated water from reactions with water and MTBSTFA. Aromatic byproducts detected here and associated with the reaction of MTBSTFA with the hydrocarbon trap Tenax TA include benzene, toluene, 1-ethynyl-3-methyl-benzene, benzoic acid, naphthalene, and diphenylmethane (*2*). Likely detection of benzene, toluene, and naphthalene in the EGA data suggests that a portion of the GCMS signals from these molecules are sourced from the sample and not the SAM hydrocarbon trap (see below).

Tenax TA adsorbent as a possible contributor to benzoic acid, benzene, toluene, and naphthalene. Another possible source of benzoic acid is the Tenax TA adsorbent present in the HC trap used to concentrate in a small volume the gases released by the sample during the pyrolysis process. Tenax TA, a polymer made of 2,6-diphenyl-*p*-phenylene oxide, releases aromatic molecules from both thermal and chemical degradation when heated to ≥300 °C (*2*). Phenol is one of the major known degradation products that was detected as its *t*-BDMS derivative in several SAM MTBSTFA GC-MS analyses as well as in laboratory experiments involving Tenax (*2*). In the presence of dioxygen, released from the decomposition of oxychlorine molecules in the sample, phenol originating from the Tenax TA adsorbent could be oxidized and transformed into benzoic acid (*3*). However, in the reducing environment in which MTBSTFA-DMF fluid wets the sample, orders of magnitude less oxygen is transferred to the Tenax TA adsorbent compared with normal pyrolysis experiments with similar volumes of sample. Moreover, with excess MTBSTFA present on the Tenax TA adsorbent, any phenol released from the trap would be rapidly derivatized, and phenol has been detected in MTBSTFA wet chemistry experiments (*4*). In addition, laboratory experiments showed that benzoic acid was detected only when Tenax TA was heated alone and that it was not detected when Tenax TA was heated in the presence of the MTBSTFA-DMF fluid (*2*). Similar experiments with TMAH-MeOH and Tenax TA have not yet been performed, but the abundance of MTBSTFA-DMF on the hydrocarbon trap from previous experiments, and the expected similar reducing influence of TMAH-MeOH suggests that although a fraction of derivatized benzoic acid could be sourced from the Tenax TA, it is unlikely to be the only contributor.

Benzene, toluene, and naphthalene are additional known byproducts of Tenax TA when 1) overheated and/or 2) reacted with MTBSTFA-DMF (*2*). The source of these three in SAM GC-MS data from Mary Anning would be indeterminant, with Tenax TA being a likely source. However, the presence of all three molecules in the evolved gas analysis (EGA), which bypasses the Tenax TA in the hydrocarbon trap, indicates that some proportion of these molecules are directly released from the organic matter in the Mary Anning sample.

1. TMAH GC-MS Analysis

EGA	M^+	Top 3 m/z	Temperature Range of Evolution	Identity
	78	78, 77, 51	375 to 500C	benzene
	91	91, 92, 65	325 to 425C	toluene
	120	105, 120, 77	300 to 375C	trimethylbenzene
	134	119, 134, 91	300 to 375C	tetramethylbenzene
	128	128, 129, 127	325 to 400C	naphthalene
	142	142, 141, 115	300 to 375C	methylnaphthalene

Number on GC1 chromatogram	M^+	Top 3 m/z	Rt (min) on SAM	Rt (sec) on SAM	Rt (min) scaled to each channel	Identity	Adjusted Rt (min) benchtop GCMS	Rt difference (min)
1	120	105, 120, 77	168.1	10088	13.5	trimethylbenzene	12.9	-0.6
BSW	147	n/a	169.0	10142	14.4	bisilylated water*	14.4	0.0
2	134	119, 134, 91	169.9	10196	15.3	tetramethylbenzene	15.6	+0.3
3	150	135, 105, 136	170.7	10239	16.0	*unknown*	*n/a*	*n/a*
4	150	150, 105, 135	171.3	10278	16.7	*unknown*	*n/a*	*n/a*
5	n/a	n/a	171.8	10307	17.1	dimethylsilanediol-2TBDMS*	nd	*n/a*
6 (Recovery std)	146	143, 147, 145	171.9	10316	17.3	fluoronaphthalene	17.3	0.0
7	128	128, 129, 127	172.2	10330	17.5	naphthalene	17.5	0.0
8	134	134, 89, 90	172.6	10355	17.9	benzothiophene	17.9	0.0

Number on GC2 chromatogram	M^+	Top 3 m/z	Rt (min) on SAM	Rt (sec) on SAM	Rt (min) scaled to each channel	Identity	Adjusted Rt (min) benchtop GCMS	Rt difference (min)
9	135	135, 134, 120	228.1	13683	7.7	*unknown*	15.8	*n/a*
10	121	120, 121, 77	229.5	13767	9.1	*unknown*	13.2	*n/a*
11	136	105, 77, 136	229.8	13790	9.5	benzoic acid methyl ester	9.3	-0.2
12	136	136, 121, 91	230.6	13837	10.3	*unknown*	nd	*n/a*
13	134	119, 134, 91	231.1	13864	10.8	*unknown*	*n/a*	*n/a*
BSW	147	n/a	231.4	13886	11.1	bisilylated water*	11.1	0.0
14	134	119, 134, 91	232.4	13942	12.1	*unknown*	nd	*n/a*
15	130	130, 129, 115	232.5	13948	12.2	dihydronaphthalene	12.7	+0.5
16	150	135, 150, 105	232.8	13965	12.4	*unknown*	nd	*n/a*
17	149	149, 148, 134	233.1	13984	12.8	*unknown*	15.8	*n/a*
6 (Recovery Std)	146	143, 147, 145	233.5	14007	13.1	fluoronaphthalene	13.9	+0.8
7	128	128, 129, 127	233.5	14012	13.2	naphthalene	13.7	+0.5
8	134	134, 89, 90	233.7	14021	13.4	benzothiophene	13.7	+0.3
18	150	150, 135, 149	234.6	14078	14.3	*unknown*	21.5	*n/a*
19	144	129, 144, 128	235.4	14125	15.1	*unknown*	nd	*n/a*
20	148	133, 148, 134	235.7	14143	15.4	*unknown*	nd	*n/a*
21	142	142, 141, 115	236.1	14163	15.7	methylnaphthalene	17.0	+1.3
5	n/a	n/a	236.3	14175	15.9	dimethylsilanediol-2TBDMS*	nd	*n/a*
22	145	145, 144, 146	237.3	14236	17.0	*unknown*	nd	*n/a*
23	168	168, 165, 167	238.2	14292	17.9	diphenylmethane*	nd	*n/a*
24	158	158, 115, 143	238.5	14311	18.2	*unknown*	nd	*n/a*

table S-2. Identification of molecules in evolved gas analysis (EGA), Gas Chromatograph (GC) 1, and Gas Chromatograph (GC) 2, with temperature range of evolution (EGA only), complementary peak number on chromatograms in Figs. 1 and 2, molecular ion, three highest abundance masses for each molecule, SAM experiment retention time, SAM retention time scaled to each channel, benchtop gas chromatograph mass spectrometry (GC-MS) retention time for comparison with candidate molecules, and difference in retention time between the SAM and benchtop comparisons, where applicable. * Denotes known SAM byproducts or background.

a. Notable results in GC1

Nine distinct peaks were identified in the GC1 chromatogram (Table 1), with the molecules trimethylbenzene (fig S-1), tetramethylbenzene (fig S-2), naphthalene (fig S-6), and benzothiophene (fig S-7) (peaks 1, 2, 7, and 8, respectively) confirmed with both benchtop retention time experiments and comparison with mass spectra from the NIST library.

Mass spectra matching several additional molecules were identified by GC-MS using GC1 (Fig. 1B) and GC2 (Fig. 1C) data, but laboratory retention time experiments conducted under SAM flight-like conditions did not confirm these identifications. Regardless, the compelling nature of the mass spectral data leads us to report on them here as plausible detections of molecules with very similar fragmentation patterns.

Peak 3, which elutes at Rt 16.0 min, is characterized by m/z 150, 135, 105, and other masses (see table S-2; fig S-3). This peak may contain 2 co-eluting molecules, and masses are consistent with molecules such as trimethylbenzendiamine (Rt = 23.6 min) and/or benzenemethanol, 4-(1-methylethyl) (Rt is ca. 3 minutes after trimethylbenzenediamine on a comparable GC column). Because the benchtop retention times are greatly offset from the flight data, we suggest these molecules at peak 3 generally consist of a benzene ring with N- and/or O-bearing functional groups.

Peak 4 represents a complex co-elution at Rt 16.7 min. Most abundant masses include m/z 150, 105, 135, and other masses (see table S-2; fig S-4). Comparisons with the NIST library were variably successful, with the molecules trimethylbenzendiamine, ethyltetramethylcyclopentadiene, and/or methyl 2-methylbenzoate identified as closest matches. The trimethylbenzendiamine Rt of 23.6 min is greatly offset from flight data, and Rt data are not available for the other two candidate molecules. Therefore, we suggest these peak 4 molecules consist of a benzene ring with N-, COOH, and/or CH_3 functional groups.

b. GC1 Comparison Chromatograms with Likely Identifications

figure S-1. Gas chromatograph comparison between SAM-Flight model spectra and closest NIST match for Peak 1. (A) Gas Chromatograph 1 Peak 1 Retention time 10088sec SAM-Flight Model spectra

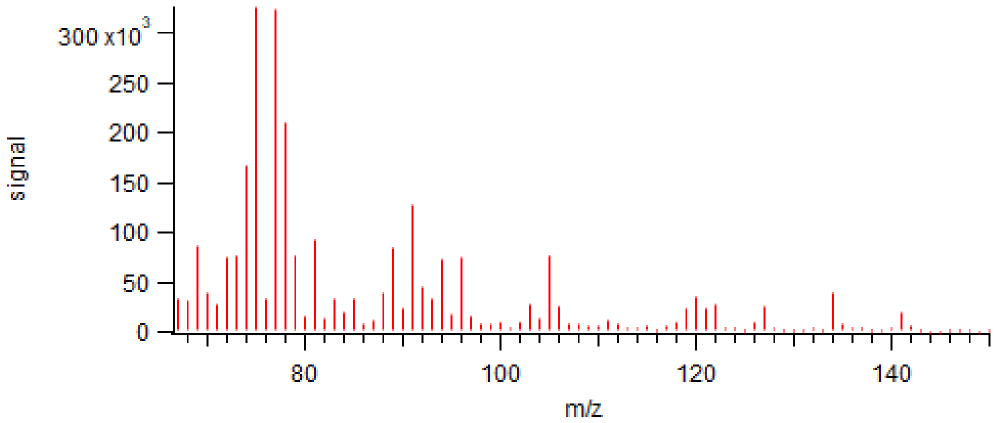

(B) Gas Chromatograph 1 Peak 1 SAM-Flight Model spectra closest NIST match: 1,3,5-Trimethylbenzene

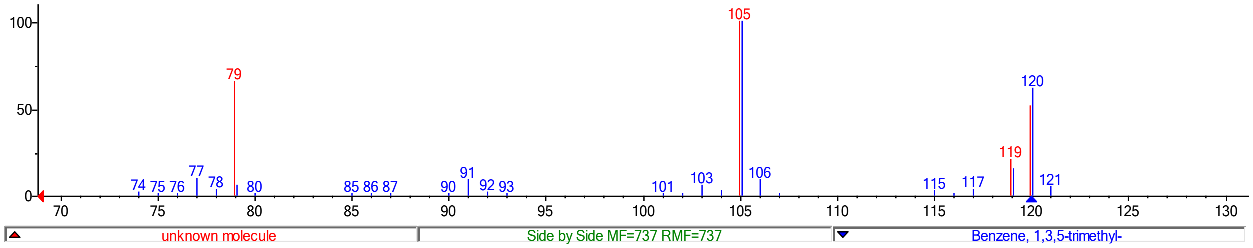

****

figure S-2. Gas chromatograph comparison between SAM-Flight model spectra and closest NIST match for Peak 2. (A) Gas Chromatograph 1 Peak 2 Retention time 10196 sec SAM-Flight Model spectra

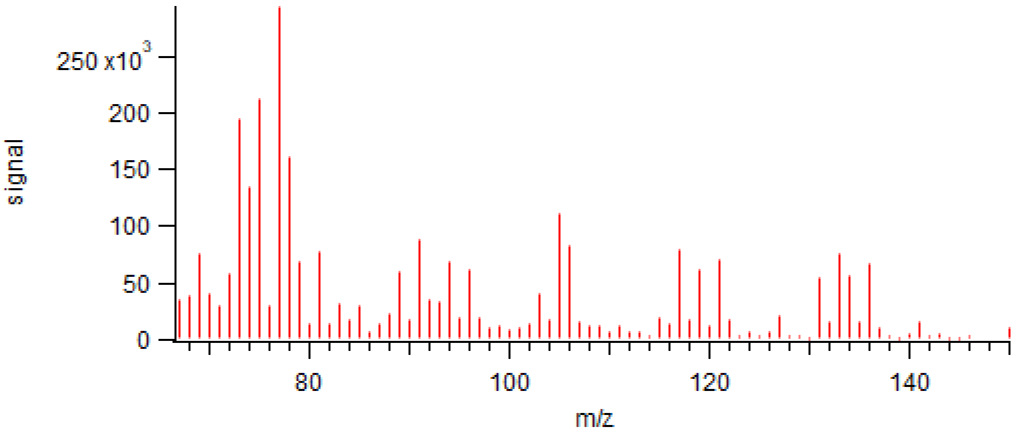

(B) Gas Chromatograph 1 Peak 2 SAM-Flight Model spectra closest NIST match: 1,2,3,5-Tetramethylbenzene

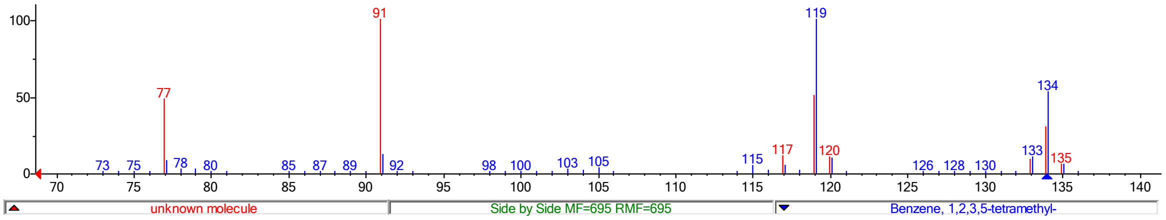

****

figure S-3. Gas chromatograph comparison between SAM-Flight model spectra and closest NIST match for Peak 3. (A) Gas Chromatograph 1 Peak 3 Retention time 10239 sec SAM-Flight Model spectra

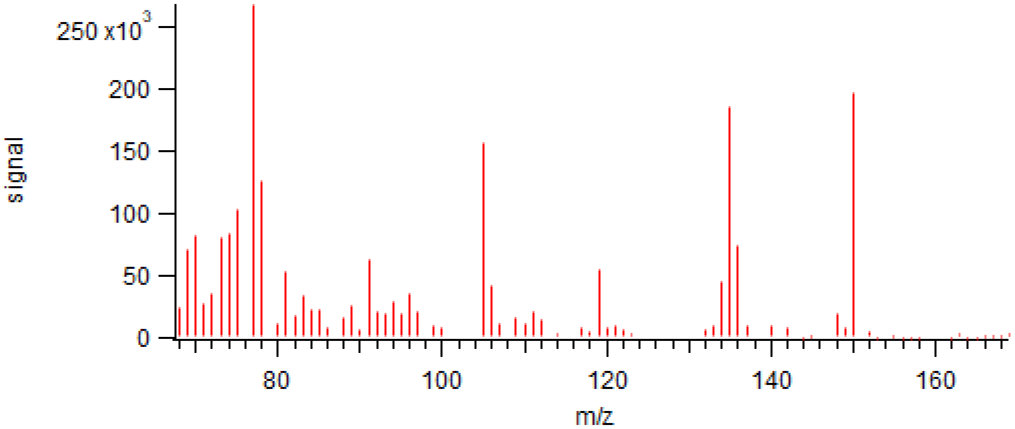

(B) Gas Chromatograph 1 Peak 3 SAM-Flight Model spectra closest NIST matches: Benzenemethanol, 4-(1-methylethyl)- AND/OR

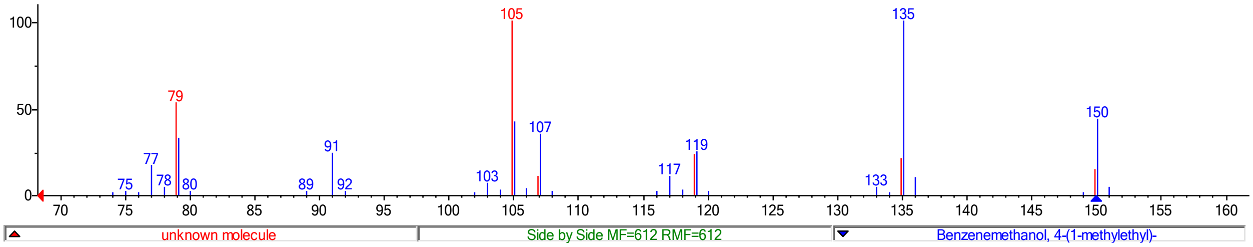

Retention time 10239 Trimethyl-1,4-benzenediamine

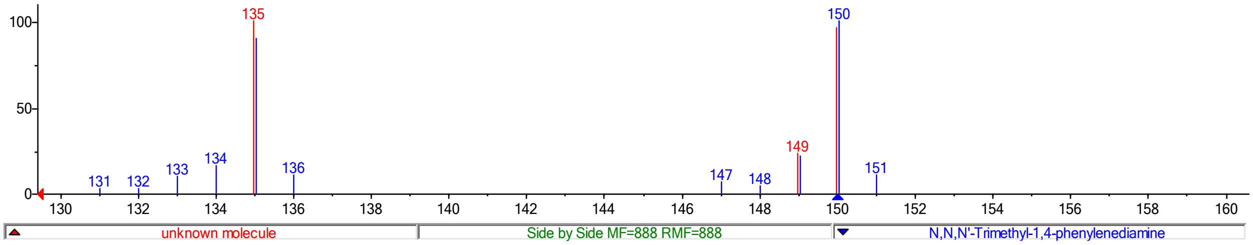

****

figure S-4. Gas chromatograph comparison between SAM-Flight model spectra and closest NIST match for Peak 4. (A) Gas Chromatograph 1 Peak 4 Retention time 10278 sec SAM-Flight Model spectra

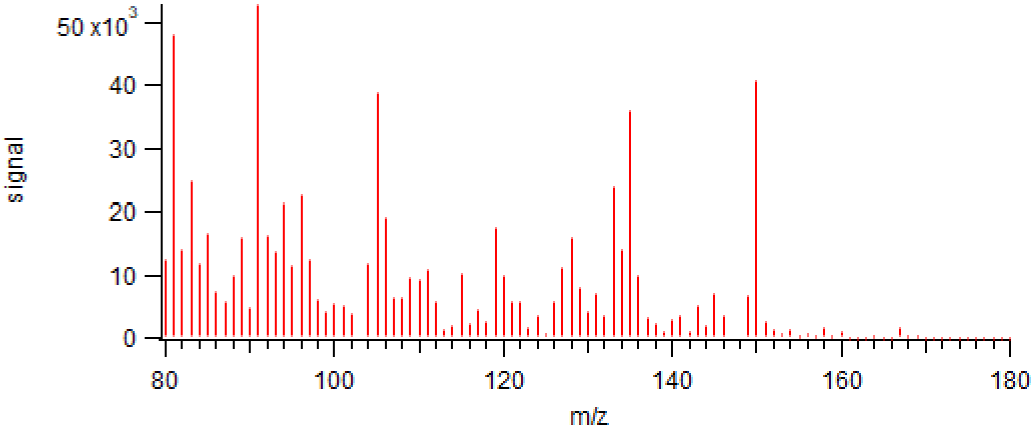

(B) Gas Chromatograph 1 Peak 4 SAM-Flight Model spectra closest NIST matches: Retention time 10278 Trimethyl-1,4-benzenediamine AND/OR

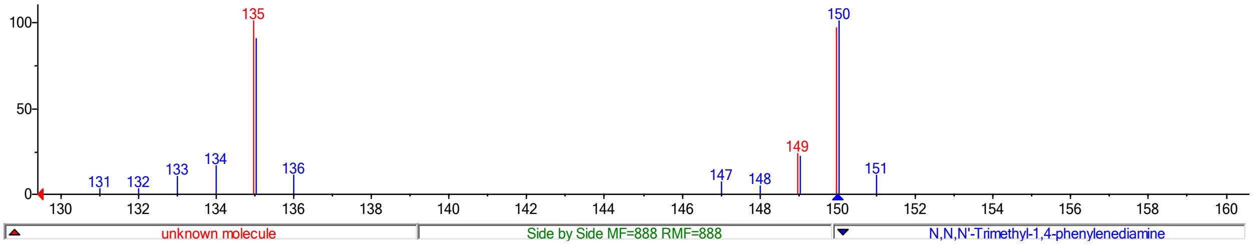

Retention time 10278 ethyltetramethylcyclopentadiene AND/OR

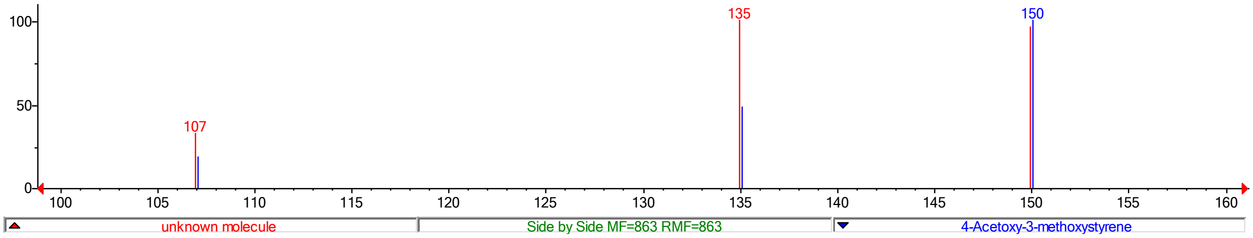

Retention time 10278 benzoic acid 2 methyl methyl ester

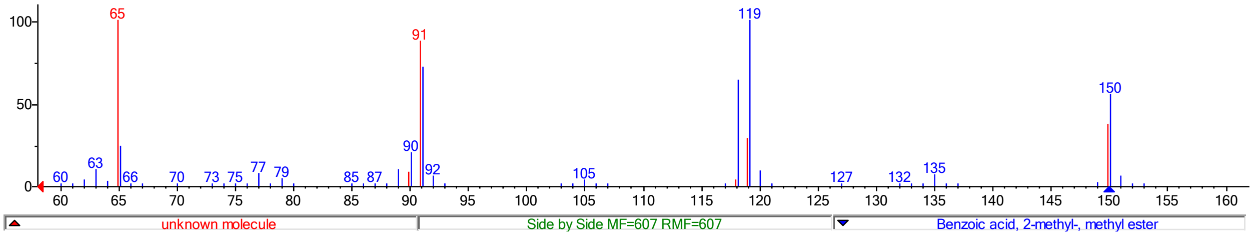

figure S-5. Gas chromatograph comparison between SAM-Flight model spectra and closest NIST match for Peak 6. (A) Gas Chromatograph 1 Peak 6 Retention time 10316 sec SAM-Flight Model spectra of Recovery Standard 1-fluoronaphthalene

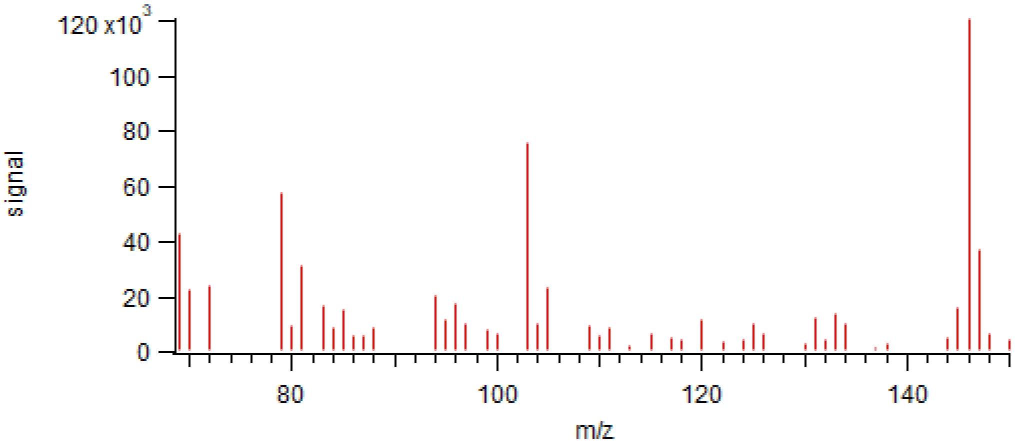

****

figure S-6. Gas chromatograph comparison between SAM-Flight model spectra and closest NIST match for Peak 7. (A) Gas Chromatograph 1 Peak 7 Retention time 10330 sec SAM-Flight Model spectra (overlap with *m/z* 146 of 1-fluoronaphthalene from Retention time10316 remains)

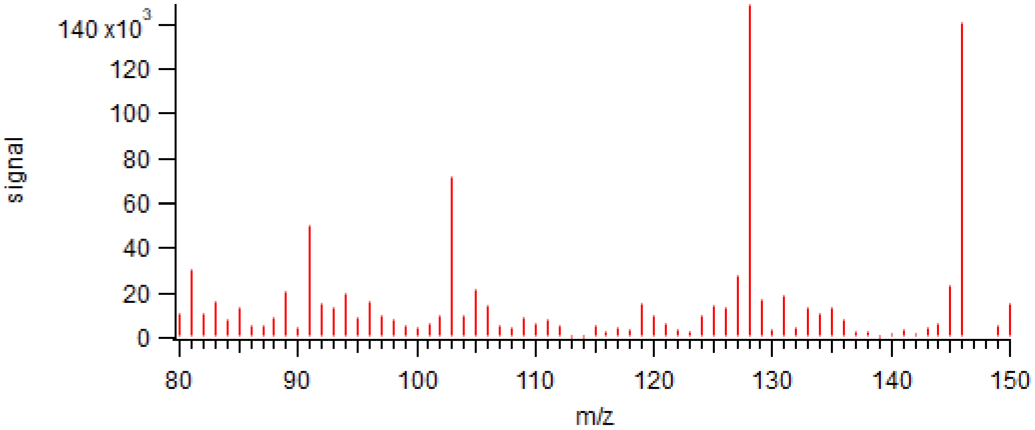

(B) Gas Chromatograph 1 Peak 7 SAM-Flight Model spectra closest NIST match: Naphthalene

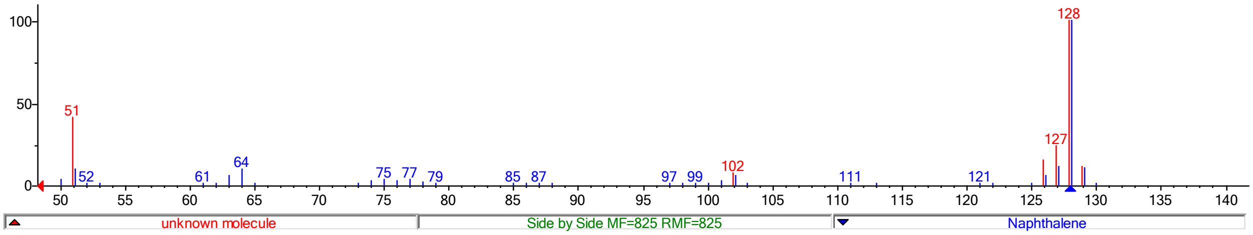

****

figure S-7. Gas chromatograph comparison between SAM-Flight model spectra and closest NIST match for Peak 8. (A) Gas Chromatograph 1 Peak 8 Retention time 10355 sec SAM-Flight Model spectra

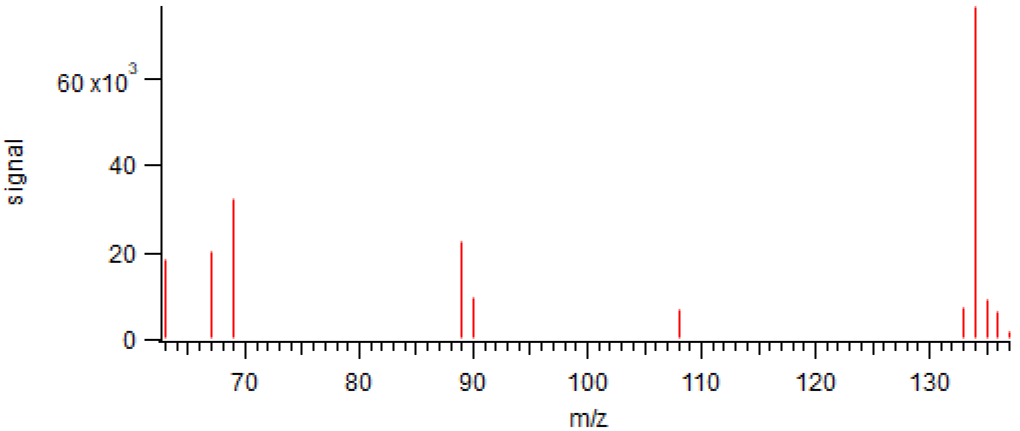

(B) Gas Chromatograph 1 Peak 8 SAM-Flight Model spectra closest NIST match: Benzothiophene

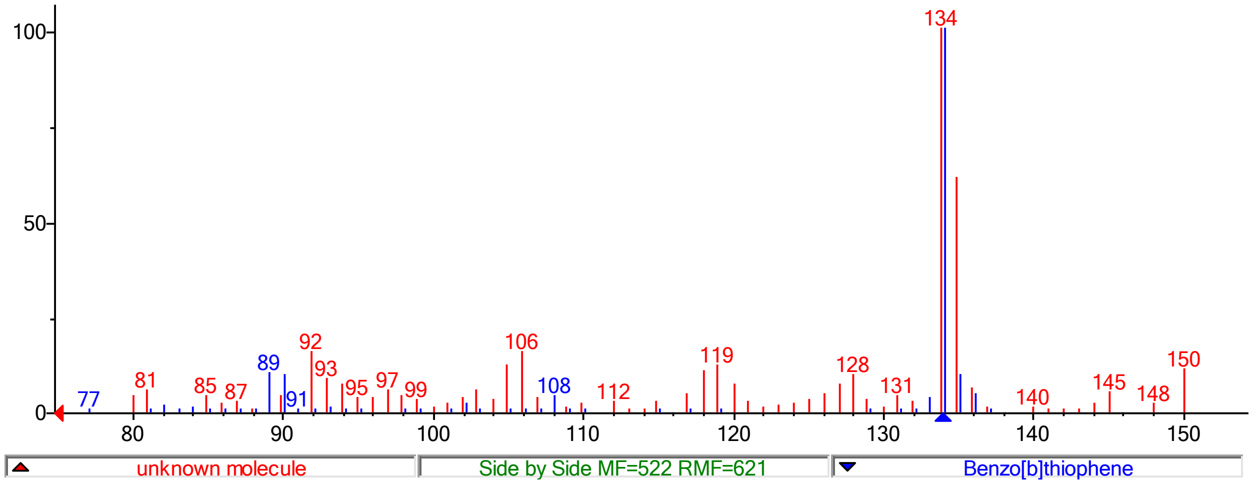

****

c. Notable results in GC2

More than 20 discrete peaks were present in the GC2 chromatogram (Table S-2), with the molecules benzoic acid methyl ester (fig S-10), dihydronaphthalene (fig S-14), naphthalene (fig S-18), benzothiophene (fig S-19), and methylnaphthalene (fig S-23) (peaks 11, 15, 7, 8, and 21 respectively) confirmed with both benchtop retention time experiments and comparison with mass spectra from the NIST library. The remaining peaks remain unidentified, but similarities in mass spectral fragments can yield insights into likely molecular identities.

Peak 9, eluting at 7.7 min, is characterized by m/z 135, 134, 120 and other masses (fig. S-8). Mass spectra are potentially consistent with benzenamine, N,N,2-trimethyl- (Rt = 15.8 min on a comparable GC column). This identification is in family with other methylated benzeneamines potentially identified in this experiment. Because the benchtop retention time is greatly offset from the flight data, we suggest this molecule generally consists of a methylated benzene ring with an amine functional group.

Peak 10 elutes at 9.1 min and is characterized by m/z 120, 121, 177 and other masses (fig. S-9). Mass spectra are quite consistent with benzenamine, N,N,dimethyl- (Rt = 13.2 min on a comparable GC column). This identification is in family with other methylated benzeneamines potentially identified in this experiment. Because the benchtop retention time is greatly offset from the flight data (and is in reverse order relative to the potential benzenamine, N,N,2-trimethyl in Peak 9), we suggest this molecule generally consists of a methylated benzene ring with an amine functional group.

Peak 12 is characterized by m/z 136, 121, 91, and 77 (fig. S-11). Comparisons with the NIST library were variably successful, with the molecules 1,4-dimethylanisole and 3,4,5-trimethylphenol identified as closest matches. We suggest this molecule consists of a multiply-methylated benzene ring with alcohol or methoxy functional group.

Peak 13 elutes at 10.8 min and is characterized by m/z 119, 134, 91 and other masses (fig. S-12). Comparisons with the NIST library were variably successful, with the molecules benzene, 1,2,4,5-tetramethyl-, O-cymene and/or benzene, 2-ethyl, 1,4-dimethyl- identified as closest matches. The benzene, 1,2,4,5-tetramethyl Rt of 9.5 min is within -1.3 minutes of the SAM Rt, making this molecule a possibility. Rt data are not available for the other two candidate molecules. Therefore, we suggest this molecule consists of a benzene ring with methyl, ethyl, and/or isopropyl groups.

Peak 14 is characterized by m/z 119, 134, 91 and other masses (fig. S-13). Comparisons with the NIST library yielded possible molecules benzene, 1,4-diethyl-, benzene, 2-ethyl, 1,4-dimethyl-, and/or benzene, 4-ethyl, 1,2-dimethyl- identified as closest matches. The benzene, 1,4-diethyl-spectral comparison is especially compelling (see SM), but Rt data are not available for this candidate molecule. We suggest this molecule consists of a single ring aromatic with methyl and/or ethyl functional groups.

Peak 16 is characterized by m/z 135, 150, 105 and other masses (fig. S-15). Comparisons with the NIST library yielded possible molecules benzene 1-methoxy-4-(1-methylethyl) or phenol, 2, 3, 5, 6-tetramethyl-identified as closest matches. Rt data are not available for these candidate molecules. We suggest this molecule consists of a single ring aromatic with methoxy, alcohol, isopropyl and/or methyl functional groups.

Peak 17 elutes at 12.8 min and is characterized by m/z 149, 148, 134 and other masses (fig. S-16). Mass spectra are potentially consistent with benzenamine, N,N,2,4-tetramethyl- (Rt = 15.8 min on a comparable GC column). Because the benchtop retention time is greatly offset from the flight data, we suggest this molecule generally consists of a methylated benzene ring with an amine functional group. This identification is in family with other methylated benzeneamines potentially identified in this experiment.

Peak 18 elutes at 14.3 min and is characterized by m/z 150, 135, 149 and other masses (fig. S-20). Mass spectra are potentially consistent with phenol, 2, 3, 5, 6-tetramethyl- (Rt not determined on a comparable GC column). A separate search within the NIH PubChem Repository yielded NNN-trimethyl-1,4-benzenediamine as a potential match. However, this molecule's Rt on the comparable GC column was greatly offset at 21.5 min relative to the candidate molecule. Therefore, we suggest this molecule generally consists of a single ring aromatic with alcohol and/or methyl functional groups.

Peak 19 is characterized by m/z 129, 144, 128 and other masses and elutes at 15.1 min (fig. S-21). NIST library searches were unproductive, so a separate search within the NIH PubChem Repository yielded naphthalene, 1,2-dihydro-3-methyl as a potential match. However, this molecule's Rt on the comparable GC column was greatly offset at 25.3 min relative to the candidate molecule at 15.1 minutes. Therefore, we suggest this molecule consists of a double ring aromatic with a methyl group. This identification is in family with other double ring aromatics confirmed or potentially identified in this experiment.

Peak 20 is characterized by m/z 133, 148, 134 and other masses (fig. S-22). Mass spectra are potentially consistent with benzene, pentamethyl-. However, this molecule's Rt on the comparable GC column was offset at 16.7 min relative to the candidate molecule at 15.4 minutes (+1.3 min Rt offset between the SAM and benchtop data). This identification is in family with other methylated benzenes confirmed or potentially identified in this experiment. We suggest this molecule consists of a multiply-methylated benzene ring.

Peak 21 elutes at 15.7 min and is characterized by m/z 142, 141, 115 and other masses (fig. S-23). Comparisons with the NIST library yielded a strong similarity to methylnaphthalene (Rt = 17.0 min on a comparable GC column). We suggest this molecule consists of a double ring aromatic with a methyl group, and despite the +1.3 min Rt offset between the SAM and benchtop data, is very likely methylnaphthalene. This identification is in family with other double ring aromatics confirmed or potentially identified in this experiment.

Peak 22 is characterized by m/z 145, 144, 146 and other masses (fig. S-24). Comparisons with the NIST library yielded a best match with dimethyl-indole. The dimethyl-indole spectral comparison is especially compelling (see below). However, this molecule's Rt on the comparable GC column was offset at 20.7 min relative to the candidate molecule at 17.0 minutes. We suggest this molecule consists of a methylated double ring aromatic with a N-heterocycle, which would be the first identification of a N-heterocycle on Mars.

Peak 23 is characterized by m/z 168, 165, 167 and other masses (fig. S-25). Comparisons with the NIST library yielded a strong similarity to diphenylmethane or 1,1-biphenyl,2-methyl-. This molecule's Rt on the comparable GC column was offset at 19.8 min relative to the candidate molecule at 17.9 minutes. Diphenylmethane is a known SAM MTBSTFA byproduct.

Peak 24 is characterized by m/z 158, 115, 143 and other masses (fig. S-26). Mass spectra are potentially consistent with 1-methoxynaphthalene (Rt not determined on a comparable GC column). This identification is in family with other double ring aromatics confirmed or potentially identified in this experiment. We suggest this molecule consists of a methoxy-bearing double ring aromatic.

d. GC2 Comparison Chromatograms with Likely Identifications

figure S-8. Gas chromatograph comparison between SAM-Flight model spectra and closest NIST match for Peak 9. (A) Gas Chromatograph 2 Peak 9 Retention time 13683 sec SAM-Flight Model spectra

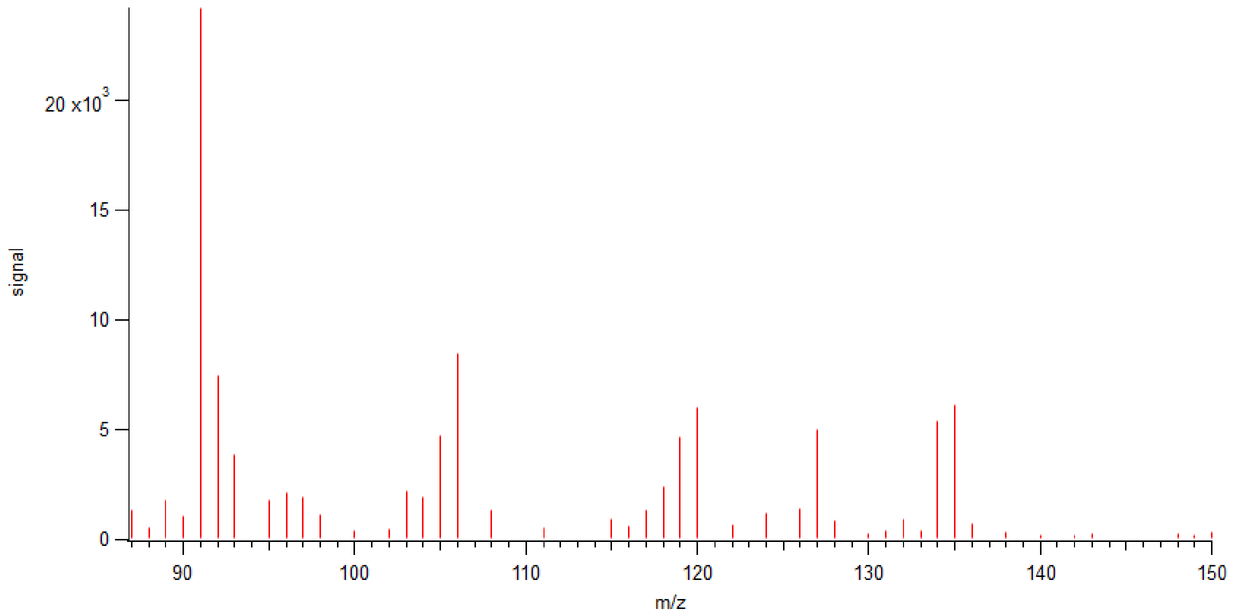

(B) Gas Chromatograph 2 Peak 9 SAM-Flight Model spectra closest NIST match: Benzenamine, N,N,2-trimethyl-

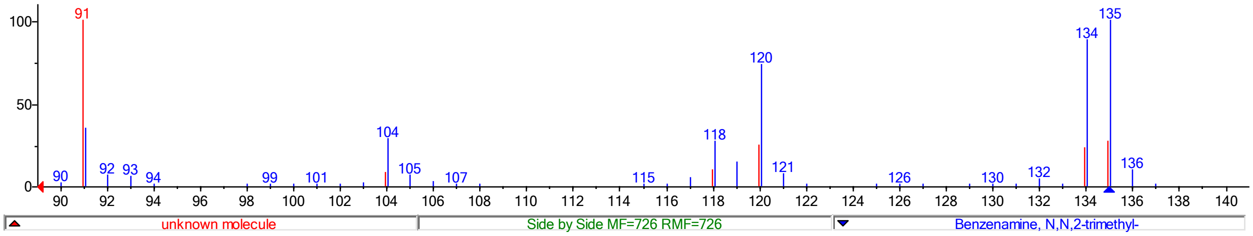

****

figure S-9. Gas chromatograph comparison between SAM-Flight model spectra and closest NIST match for Peak 10. (A) Gas Chromatograph 2 Peak 10 Retention time 13767 sec SAM-Flight Model spectra

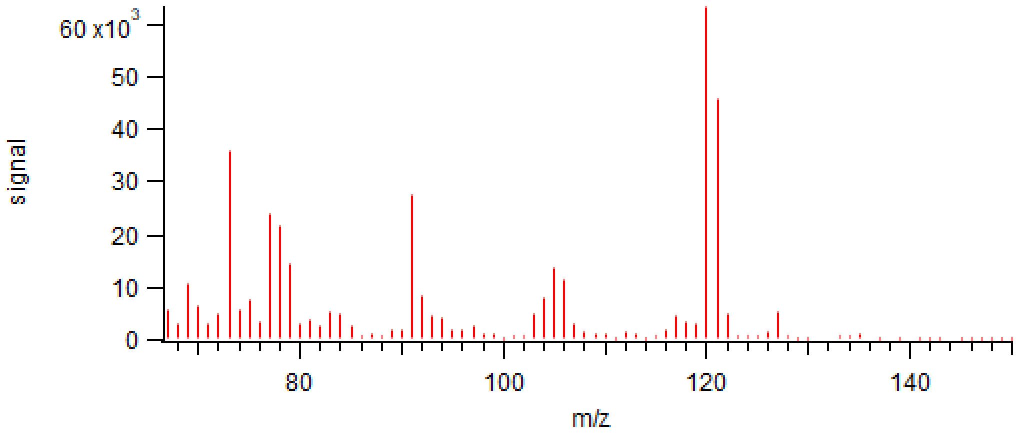

(B) Gas Chromatograph 2 Peak 10 SAM-Flight Model spectra closest NIST match: Benzenamine, N,N-dimethyl-

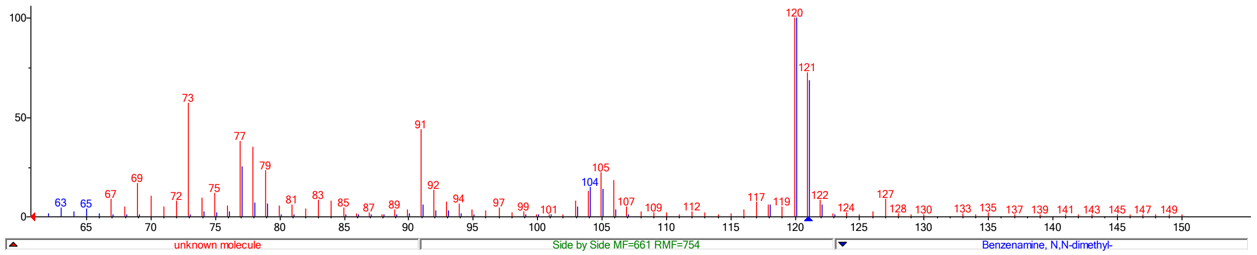

****

figure S-10. Gas chromatograph comparison between SAM-Flight model spectra and closest NIST match for Peak 11. (A) Gas Chromatograph 2 Peak 11 Retention time 13790 sec SAM-Flight Model spectra

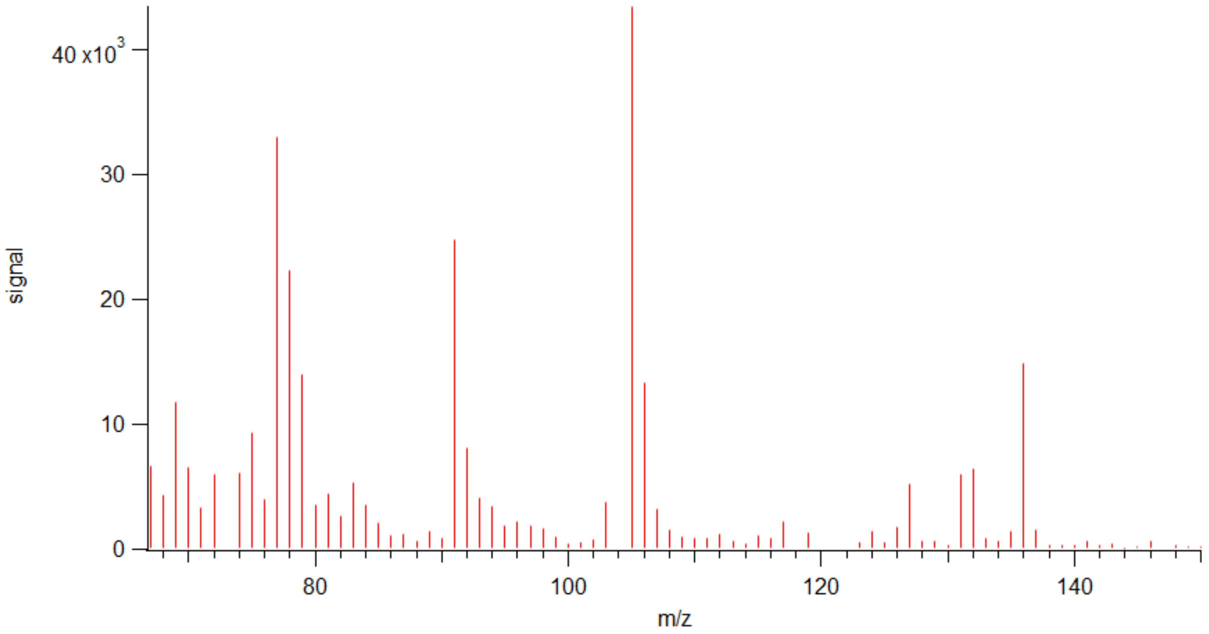

(B) Gas Chromatograph 2 Peak 11 SAM-Flight Model spectra closest NIST match: Benzoic acid methyl ester

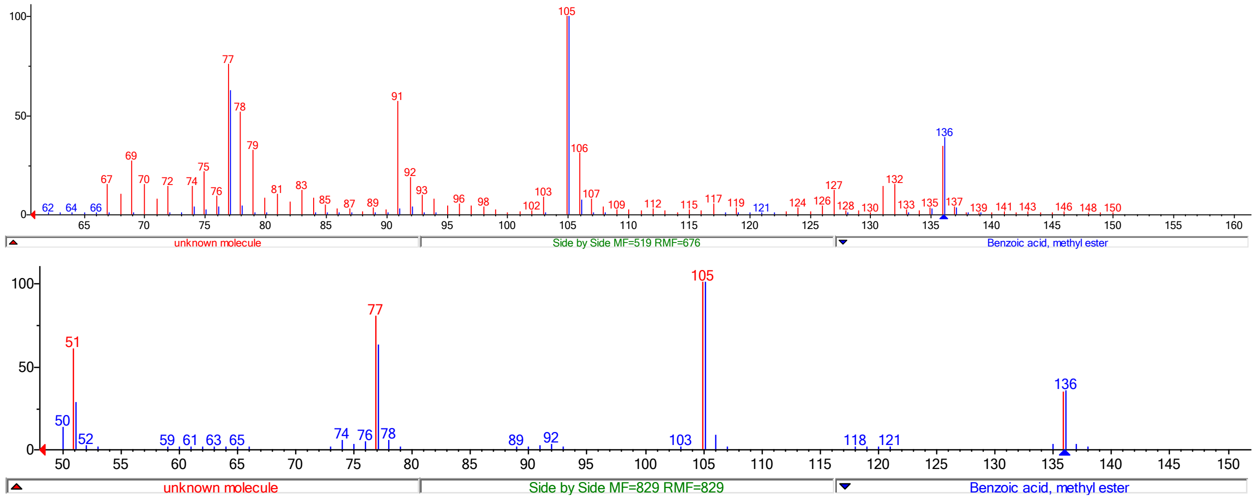

****

figure S-11. Gas chromatograph comparison between SAM-Flight model spectra and closest NIST match for Peak 12. (A) Gas Chromatograph 2 Peak 12 Retention time 13837 sec SAM-Flight Model spectra

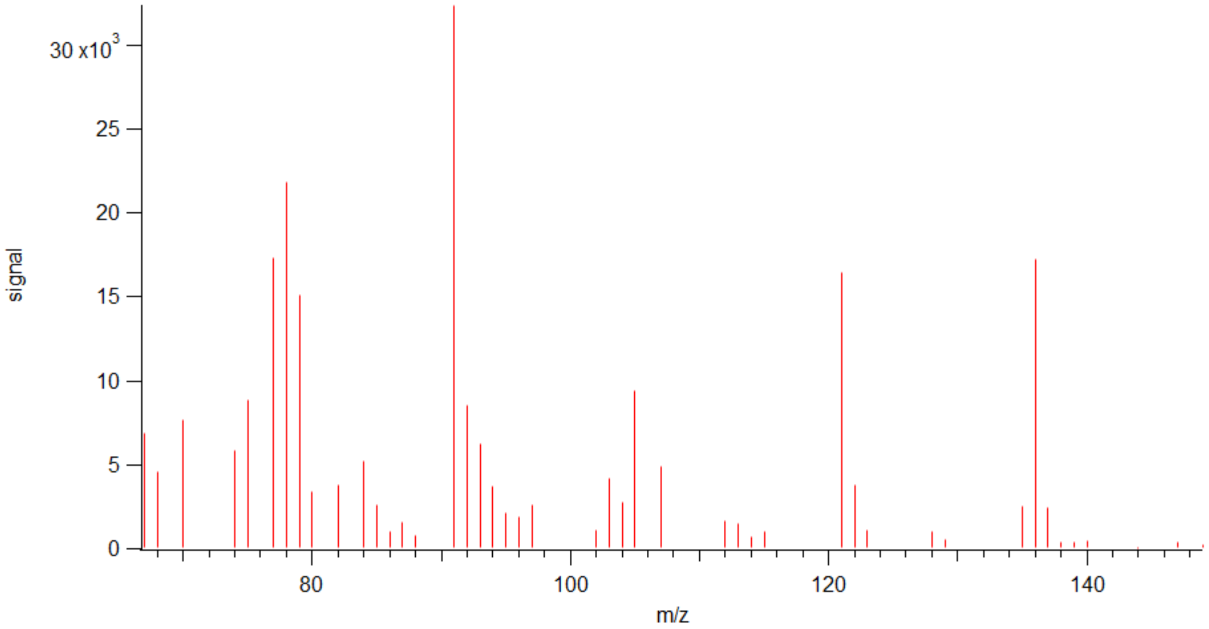

(B) Gas Chromatograph 2 Peak 12 SAM-Flight Model spectra closest NIST matches: 1,4-Dimethylanisole AND/OR

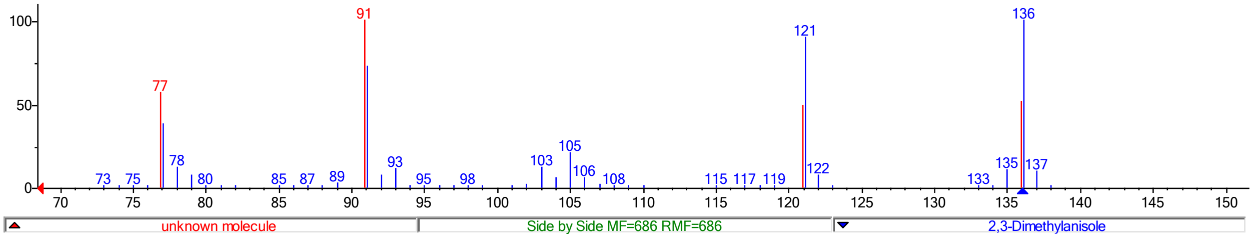

3, 4, 5-trimethylphenol

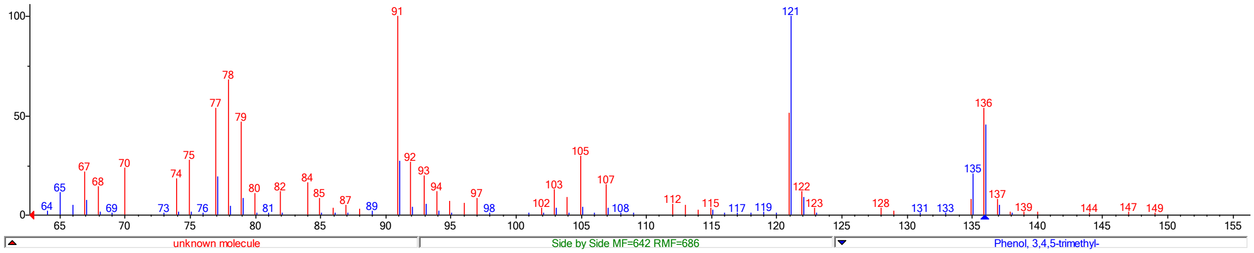

****

figure S-12. Gas chromatograph comparison between SAM-Flight model spectra and closest NIST match for Peak 13. (A) Gas Chromatograph 2 Peak 13 Retention time 13864 sec SAM-Flight Model spectra

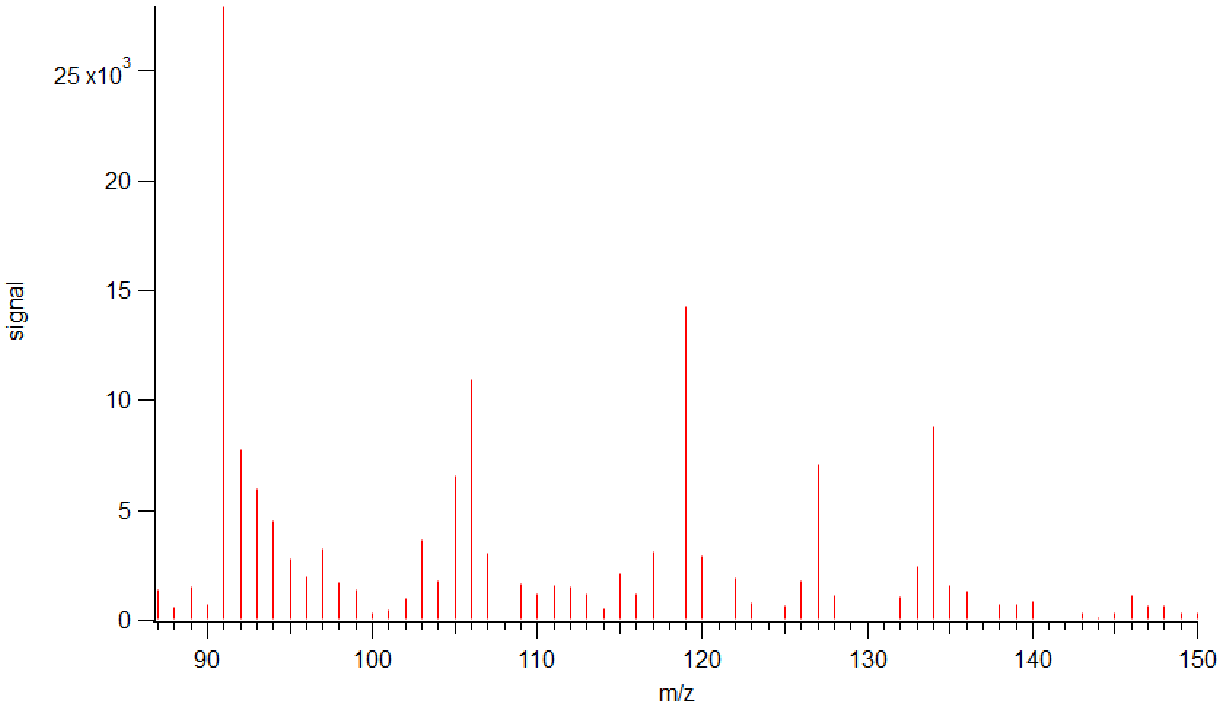

(B) Gas Chromatograph 2 Peak 13 SAM-Flight Model spectra closest NIST matches: Benzene, 1,2,4,5-tetramethyl- AND/OR

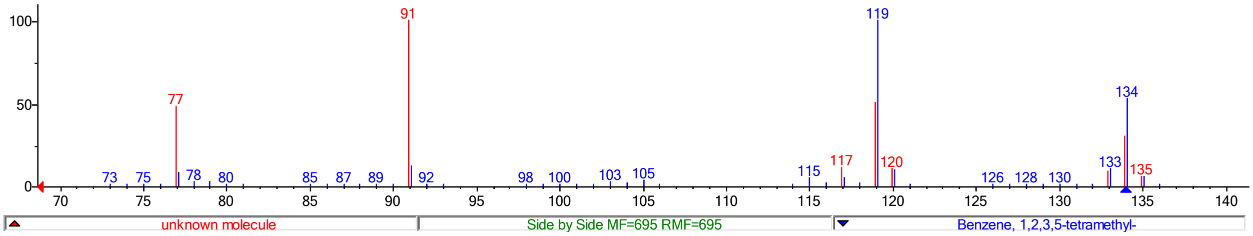

o-Cymene AND/OR

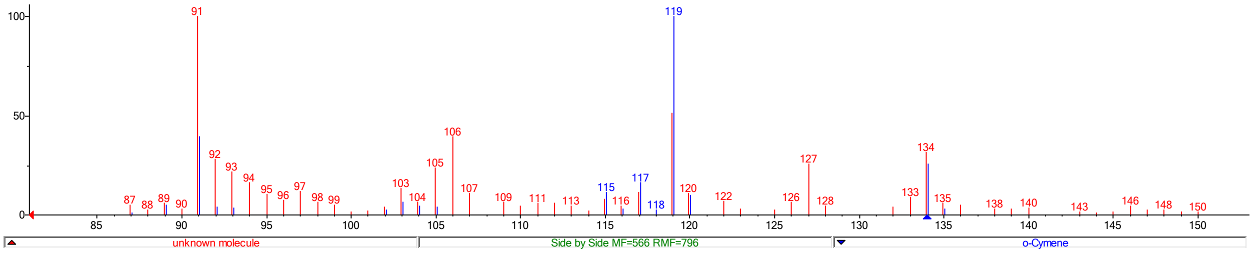

Benzene, 2-ethyl, 1,4-dimethyl

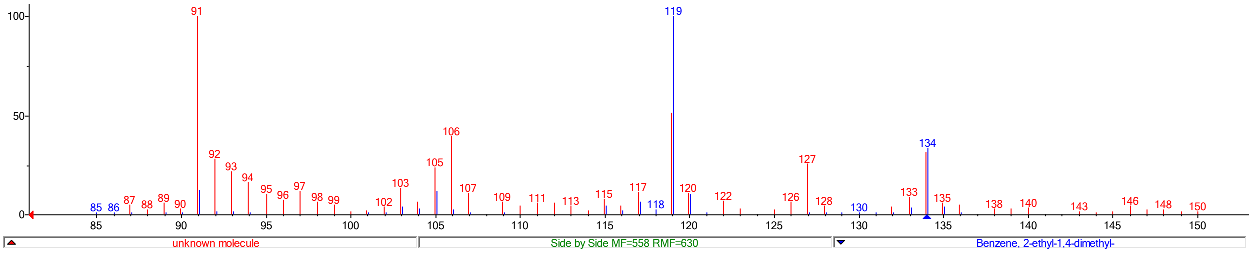

figure S-13. Gas chromatograph comparison between SAM-Flight model spectra and closest NIST match for Peak 14. (A) Gas Chromatograph 2 Peak 14 Retention time 13942 sec SAM-Flight Model spectra

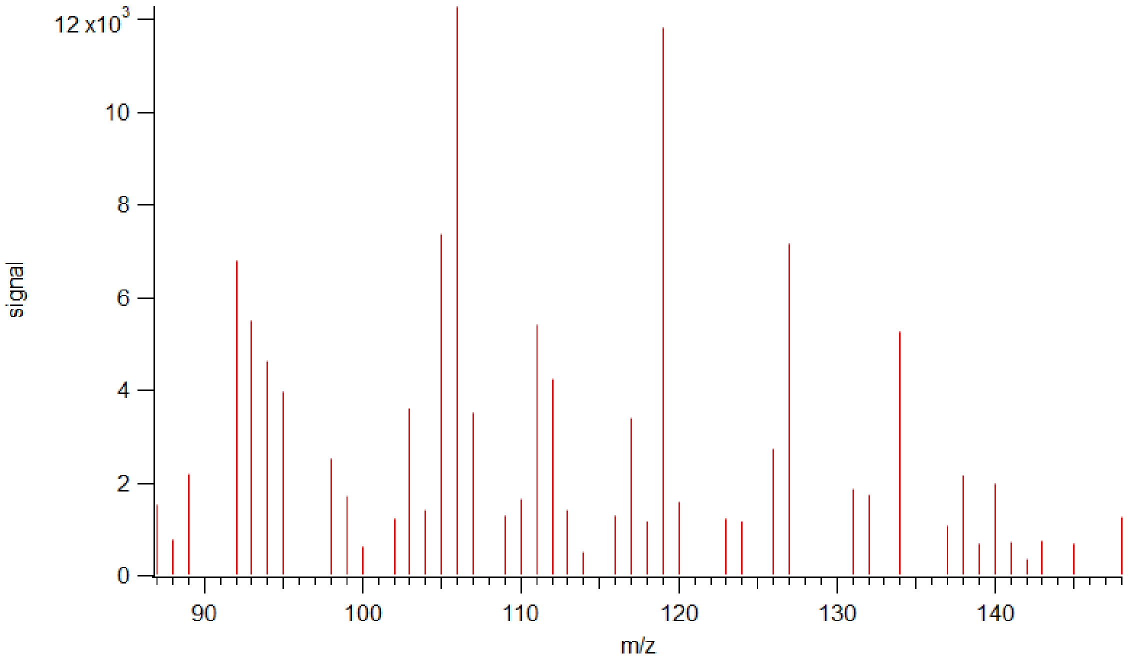

(B) Gas Chromatograph 2 Peak 14 SAM-Flight Model spectra closest NIST matches: Benzene, 1,4-diethyl- AND/OR

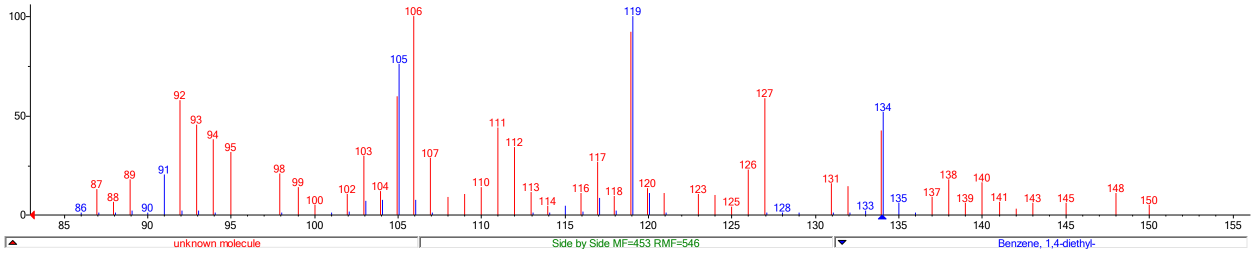

Benzene, 2-ethyl-1,4-dimethyl- AND/OR

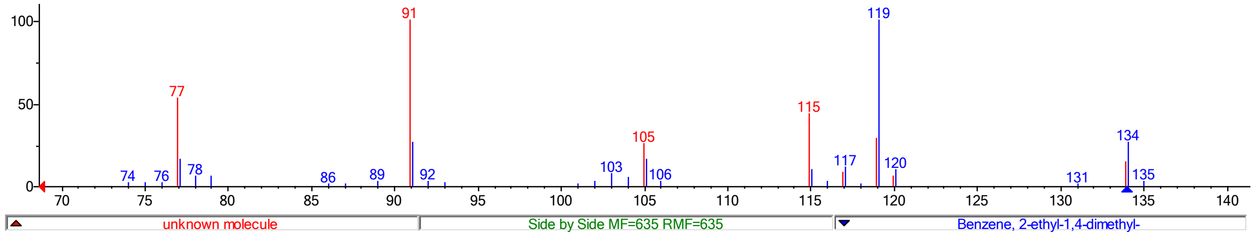

Benzene, 4-ethyl-1,2-dimethyl-

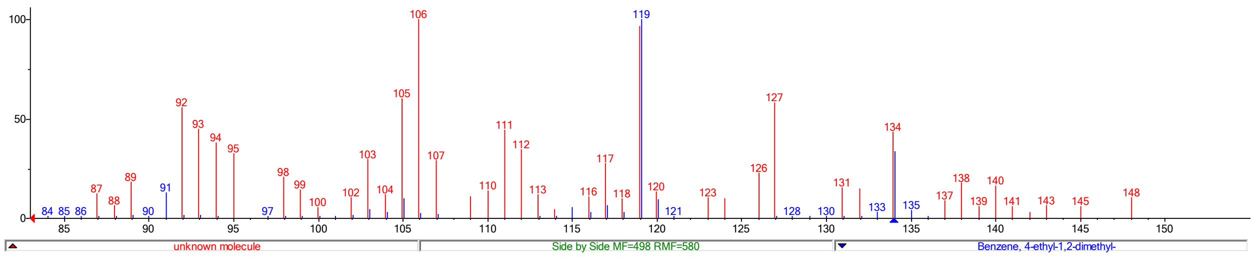

figure S-14. Gas chromatograph comparison between SAM-Flight model spectra and closest NIST match for Peak 15. (A) Gas Chromatograph 2 Peak 15 Retention time 13948 sec SAM-Flight Model spectra

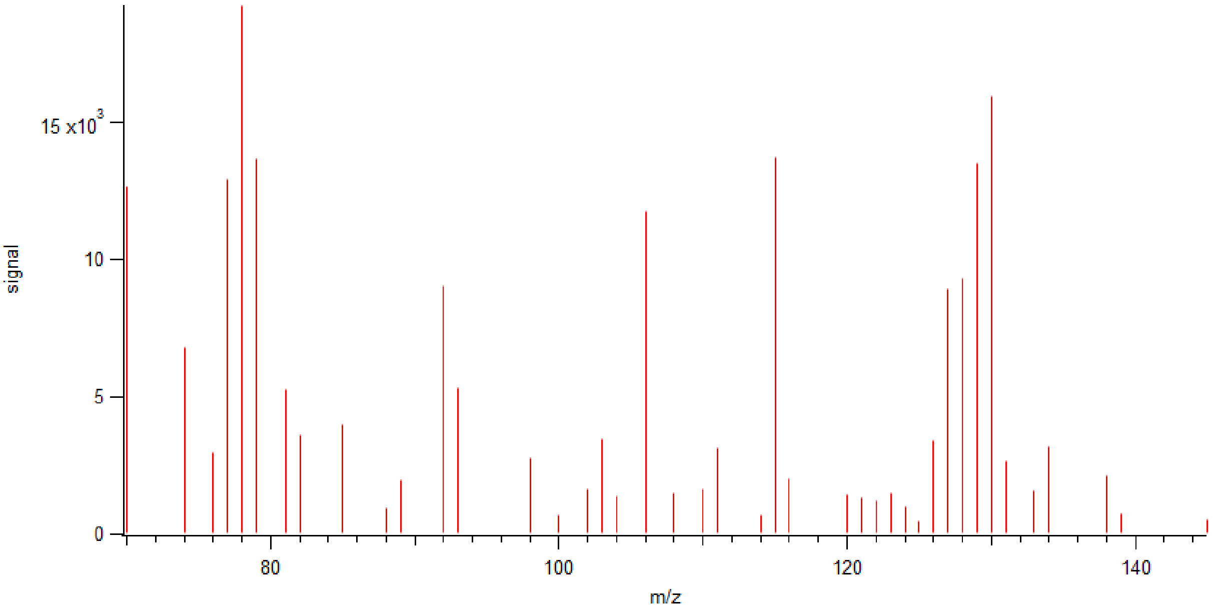

(B) Gas Chromatograph 2 Peak 15 SAM-Flight Model spectra closest NIST match: Naphthalene, 1,2-dihydro-

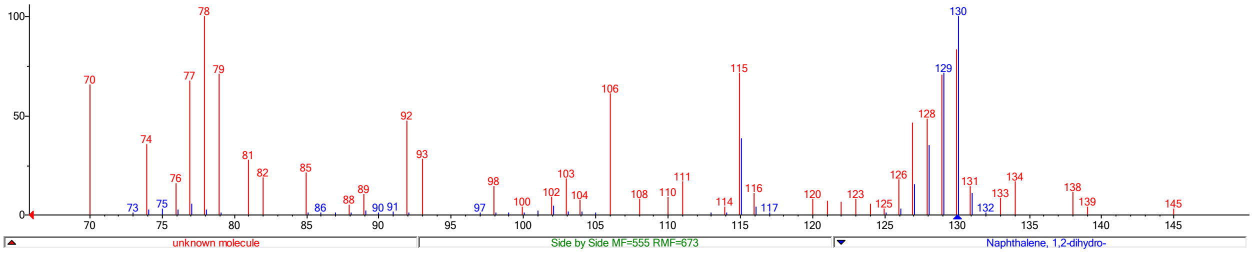

****

figure S-15. Gas chromatograph comparison between SAM-Flight model spectra and closest NIST match for Peak 16. (A) Gas Chromatograph 2 Peak 16 Retention time 13965 sec SAM-Flight Model spectra

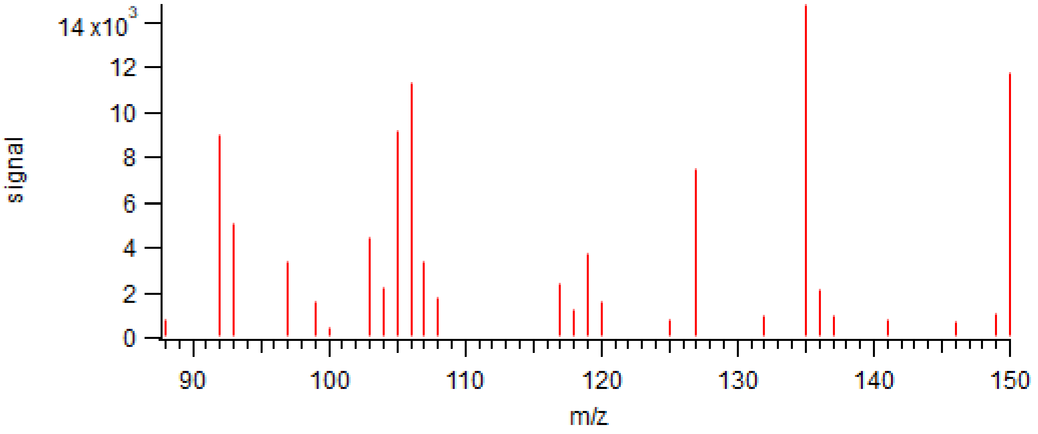

(B) Gas Chromatograph 2 Peak 16 SAM-Flight Model spectra closest NIST matches: benzene 1-methoxy-4-(1-methylethyl) AND/OR

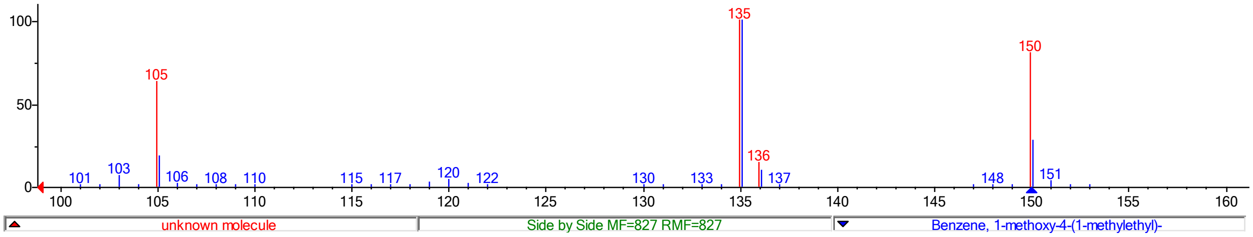

phenol, 2, 3, 5, 6-tetramethyl-

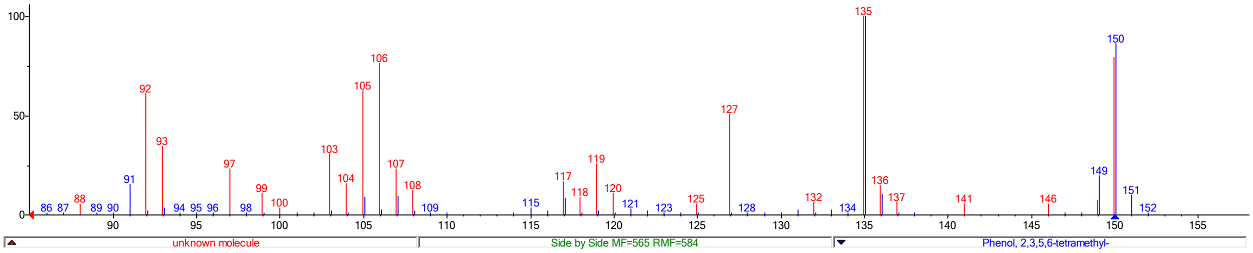

****

figure S-16. Gas chromatograph comparison between SAM-Flight model spectra and closest NIST match for Peak 17. (A) Gas Chromatograph 2 Peak 17 Retention time 13984 sec SAM-Flight Model spectra

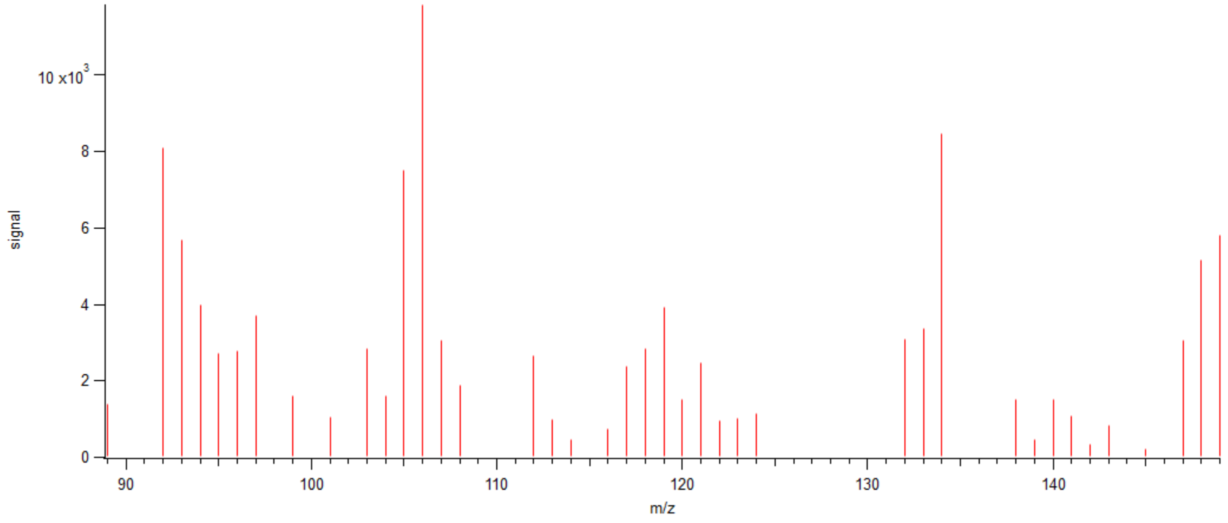

(B) Gas Chromatograph 2 Peak 17 SAM-Flight Model spectra closest NIST match: NN2,4-tetramethylbenzenamine

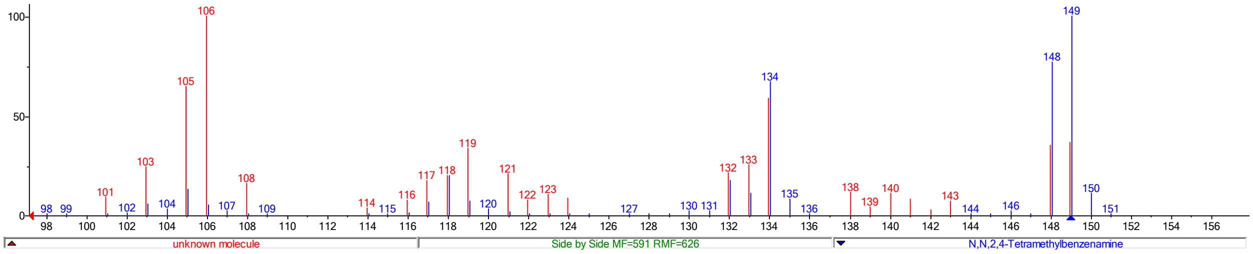

****

figure S-17. Gas chromatograph comparison between SAM-Flight model spectra and closest NIST match for Peak 6. (A) Gas Chromatograph 2 Peak 6 Retention time 14007 sec SAM-Flight Model spectra of Recovery Standard 1-fluoronaphthalene

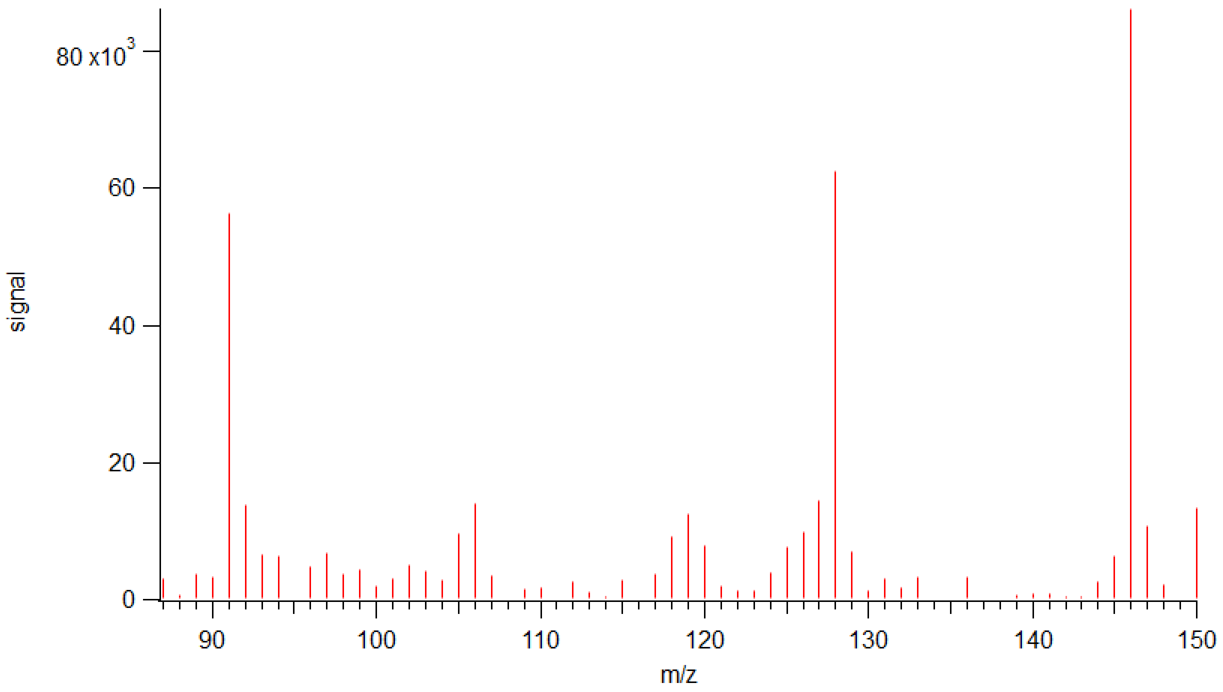

(B) Gas Chromatograph 2 Peak 6 SAM-Flight Model spectra closest NIST match: 1-fluoronaphthalene (Recovery Standard)

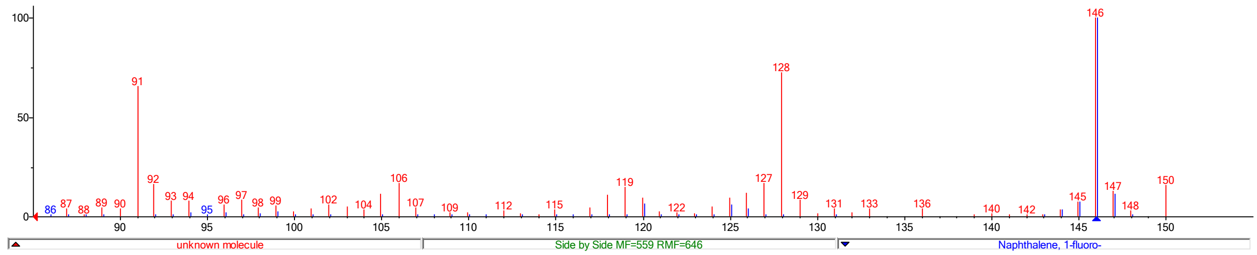

****

figure S-18. Gas chromatograph comparison between SAM-Flight model spectra and closest NIST match for Peak 7. (A) Gas Chromatograph 2 Peak 7 Retention time 14012 sec SAM-Flight Model spectra

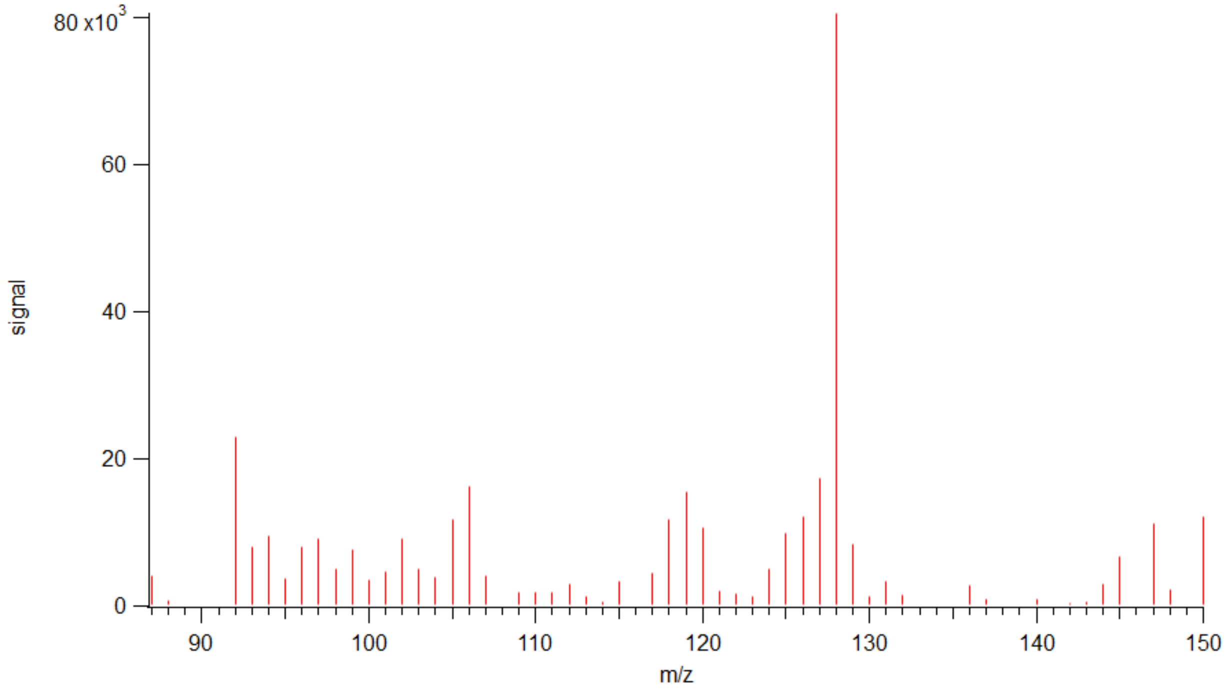

(B) Gas Chromatograph 2 Peak 7 SAM-Flight Model spectra closest NIST matches: Naphthalene

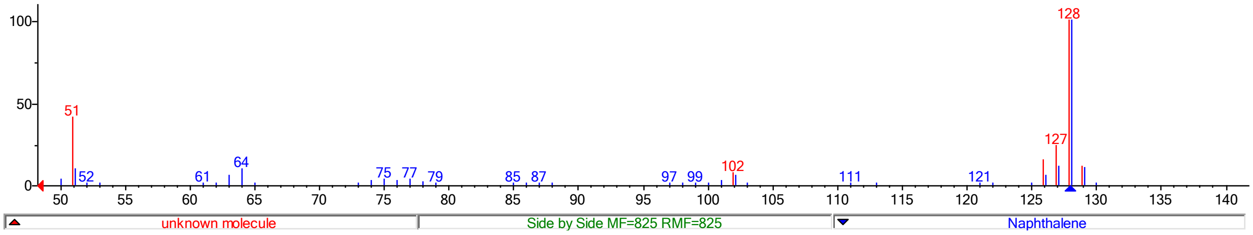

Naphthalene

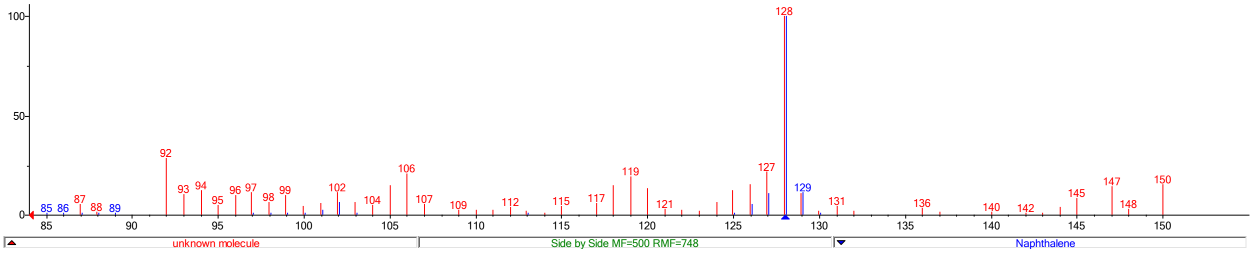

****

figure S-19. Gas chromatograph comparison between SAM-Flight model spectra and closest NIST match for Peak 8. (A) Gas Chromatograph 2 Peak 8 Retention time 14021 sec SAM-Flight Model spectra

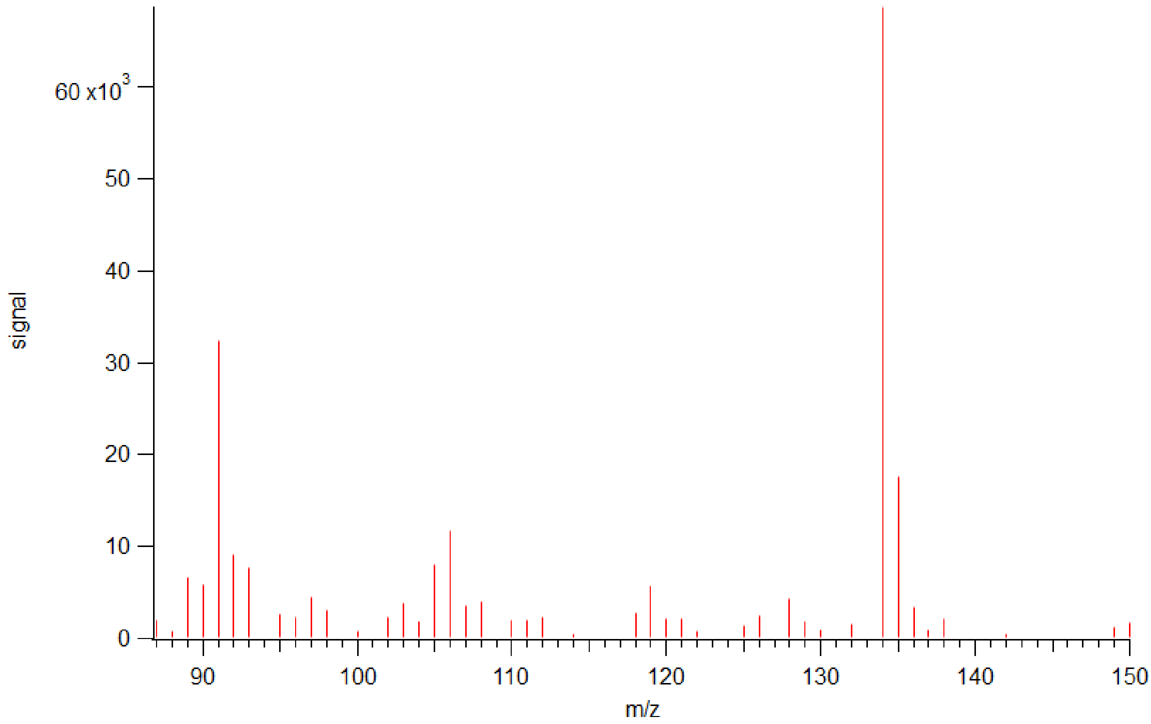

(B) Gas Chromatograph 2 Peak 8 SAM-Flight Model spectra closest NIST matches: Benzothiophene

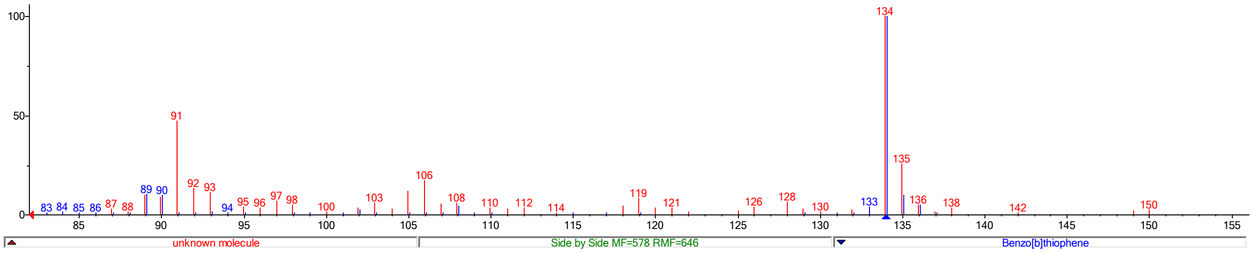

Benzothiophene

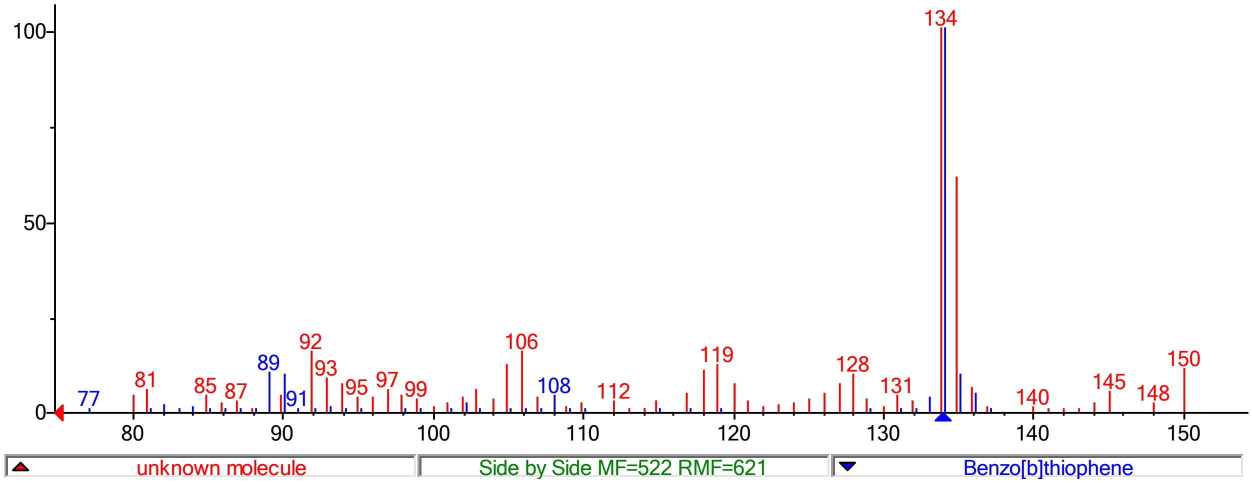

figure S-20. Gas chromatograph comparison between SAM-Flight model spectra and closest NIST match for Peak 18. (A) Gas Chromatograph 2 Peak 18 Retention time 14078 sec SAM-Flight Model spectra

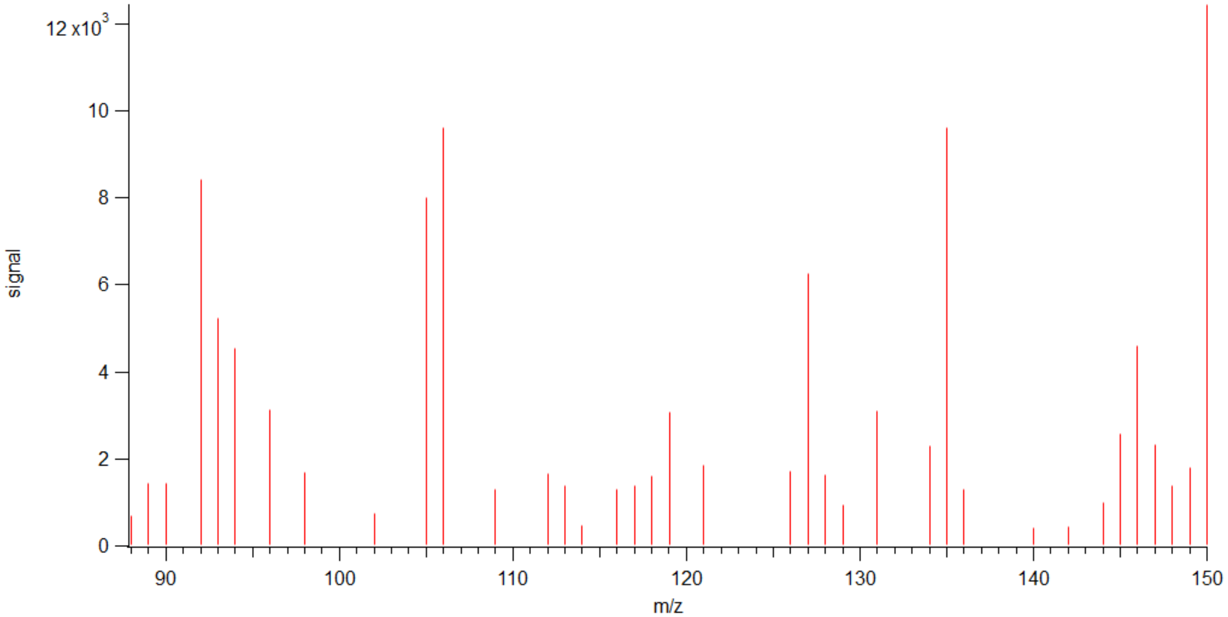

(B) Gas Chromatograph 2 Peak 18 SAM-Flight Model spectra closest NIST matches: phenol, 2, 3, 5, 6-tetramethyl- AND/OR

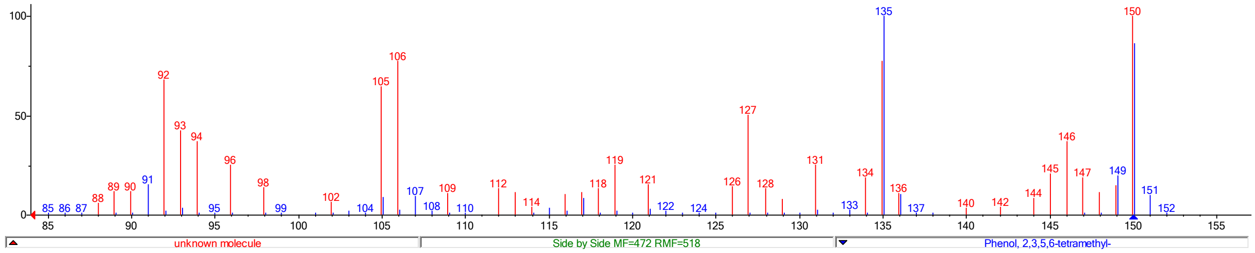

NNN-trimethyl-1,4-benzenediamine (no match in NIST library)

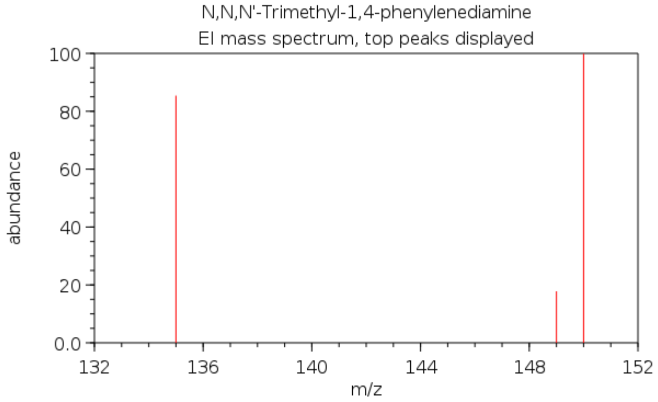

© 2014 by the U.S. Secretary of Commerce.

https://pubchem.ncbi.nlm.nih.gov/compound/N_N_N_-Trimethyl-1_4-benzenediamine

****

figure S-21. Gas chromatograph comparison between SAM-Flight model spectra and closest NIST match for Peak 19. (A) Gas Chromatograph 2 Peak 19 Retention time 14125 sec SAM-Flight Model spectra

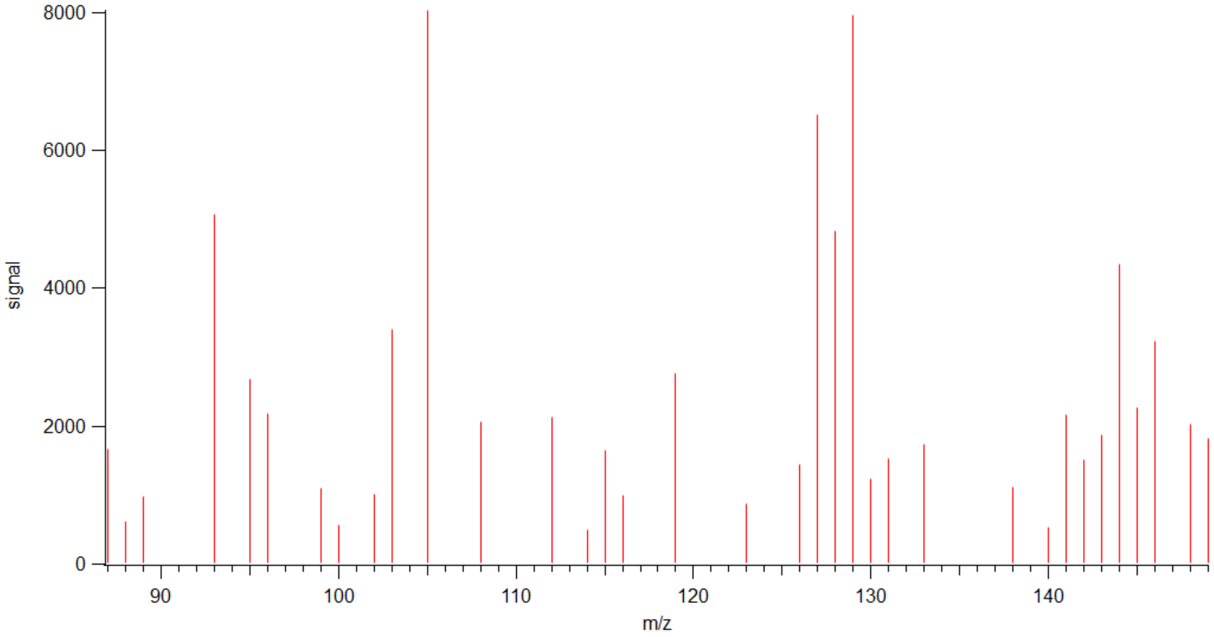

(B) Gas Chromatograph 2 Peak 19 SAM-Flight Model spectra closest NIST match: naphthalene, 1,2-dihydro-3-methyl

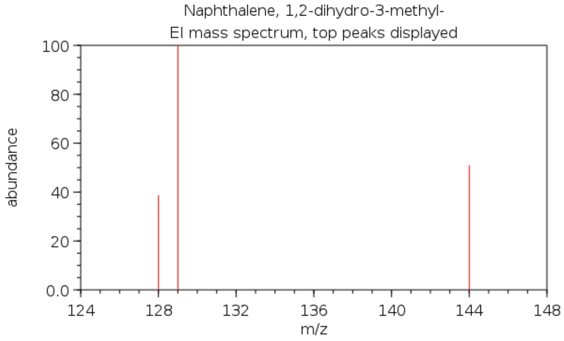

© 2014 by the U.S. Secretary of Commerce.

https://pubchem.ncbi.nlm.nih.gov/compound/3-Methyl-1_2-dihydronaphthalene#section=GC-MS

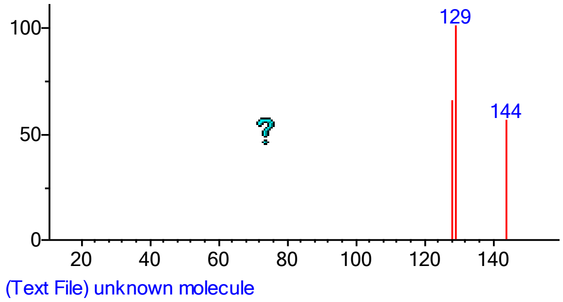

****

figure S-22. Gas chromatograph comparison between SAM-Flight model spectra and closest NIST match for Peak 20. (A) Gas Chromatograph 2 Peak 20 Retention time 14143 sec SAM-Flight Model spectra

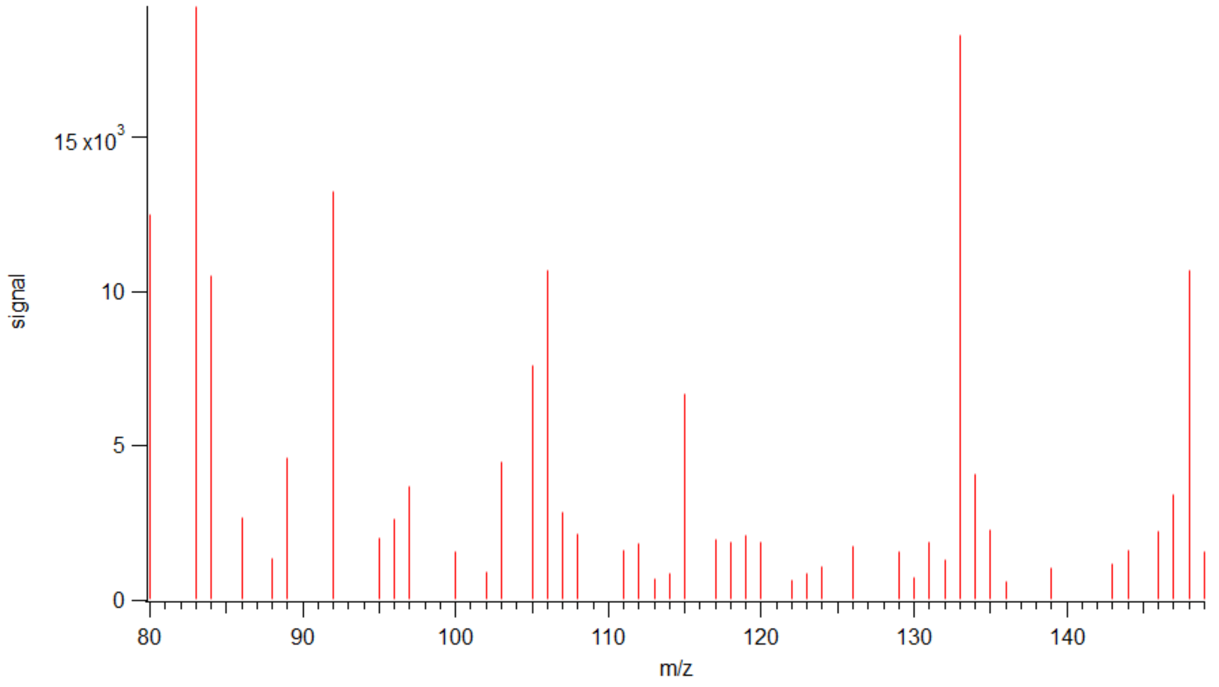

(B) Gas Chromatograph 2 Peak 20 SAM-Flight Model spectra closest NIST match: Benzene, pentamethyl

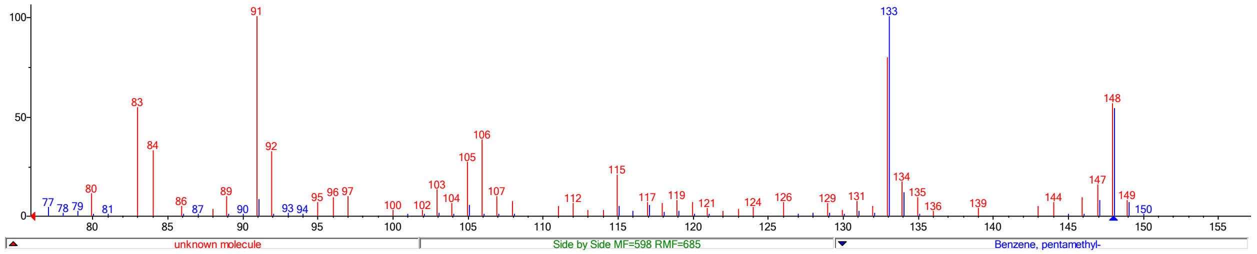

****

figure S-23. Gas chromatograph comparison between SAM-Flight model spectra and closest NIST match for Peak 21. (A) Gas Chromatograph 2 Peak 21 Retention time 14163 sec SAM-Flight Model spectra

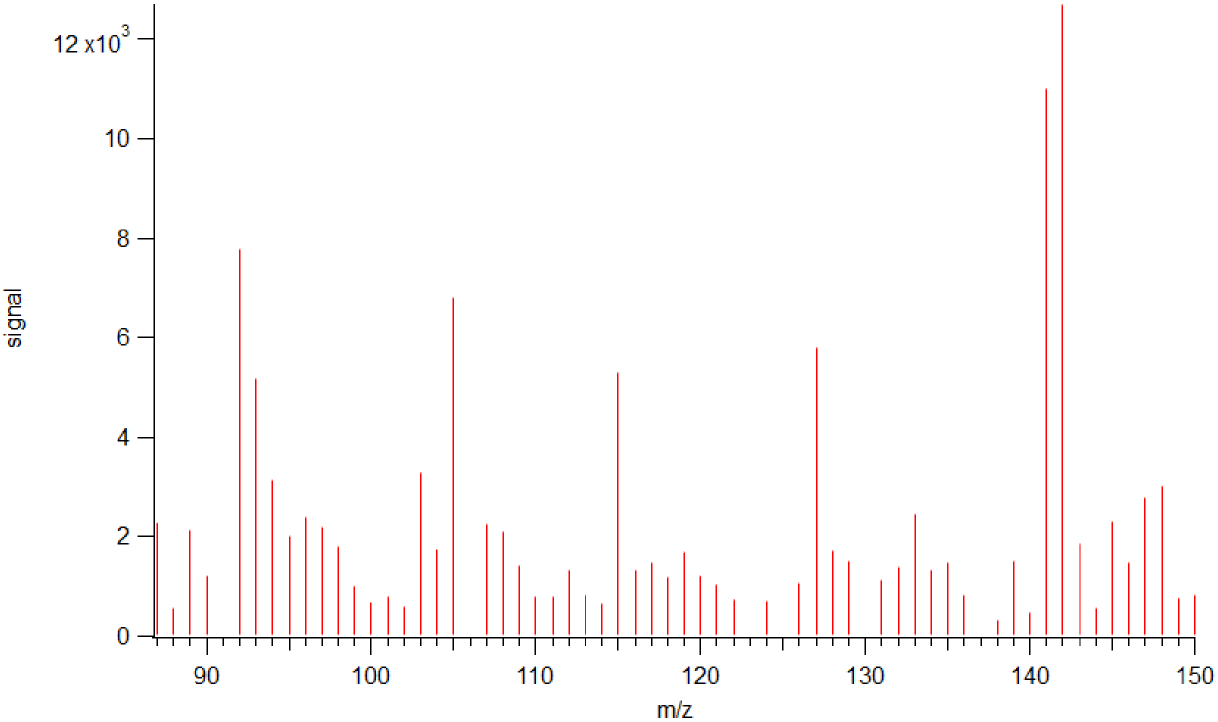

(B) Gas Chromatograph 2 Peak 21 SAM-Flight Model spectra closest NIST match: Retention time 14163 2-Methylnaphthalene OR

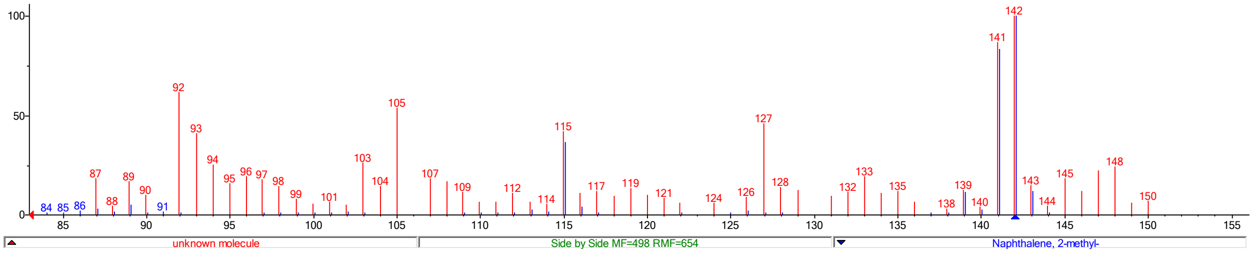

Retention time 14163 1-Methylnaphthalene

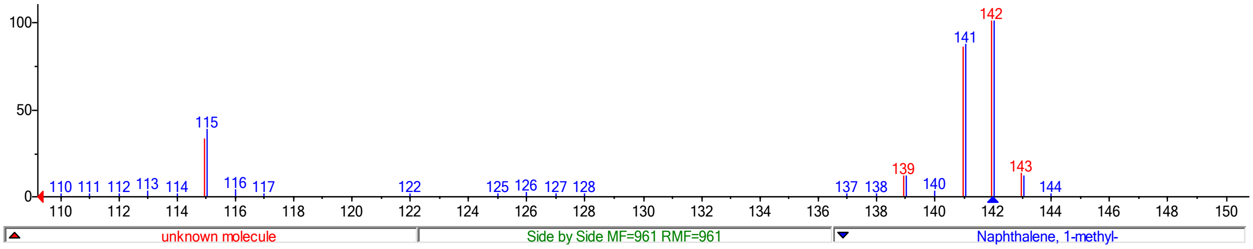

****

figure S-24. Gas chromatograph comparison between SAM-Flight model spectra and closest NIST match for Peak 22. (A) Gas Chromatograph 2 Peak 22 Retention time 14236 sec SAM-Flight Model spectra

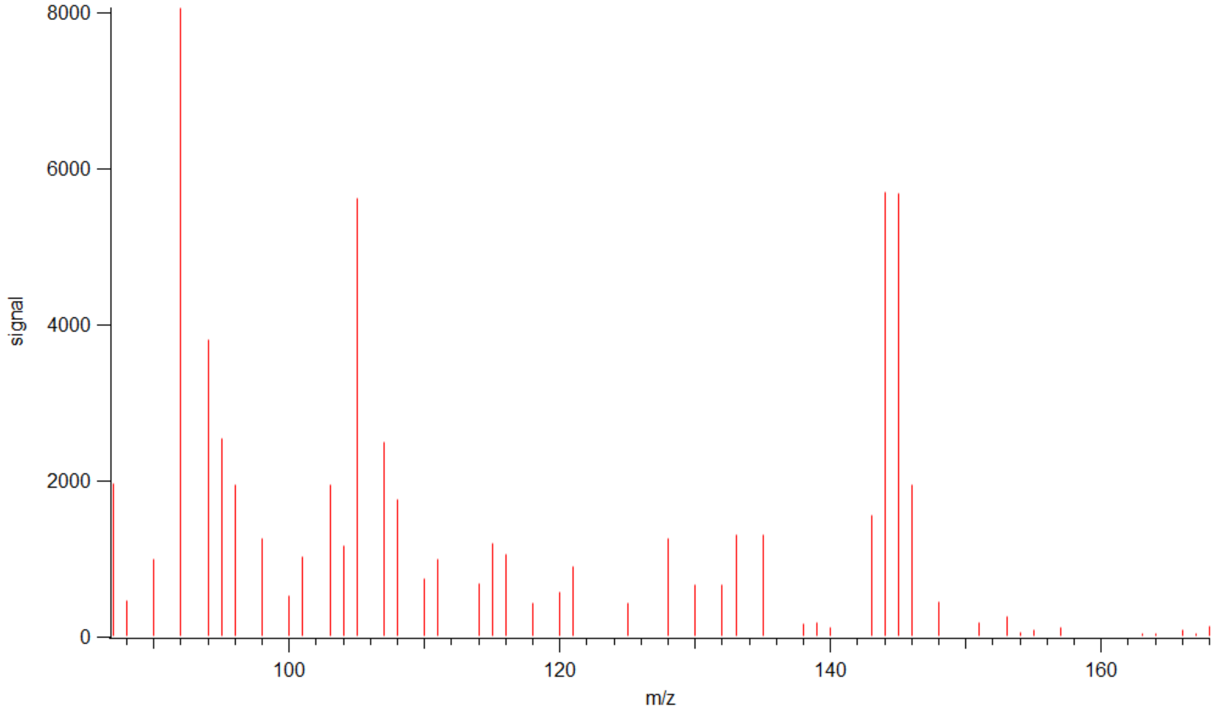

(B) Gas Chromatograph 2 Peak 22 SAM-Flight Model spectra closest NIST match: Dimethyl-indole

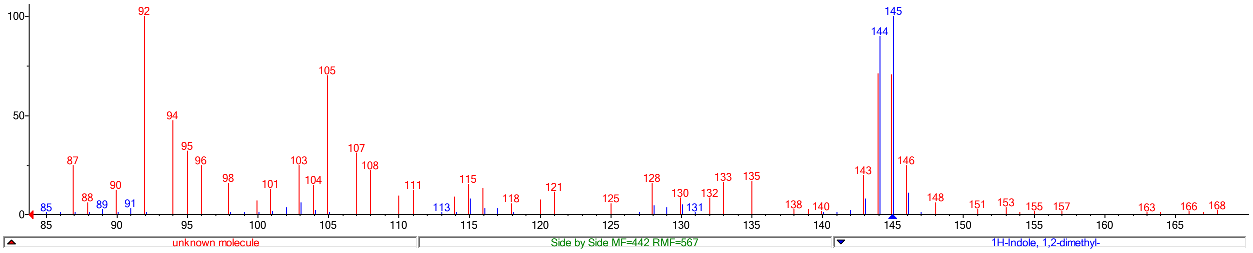

Dimethyl-indole

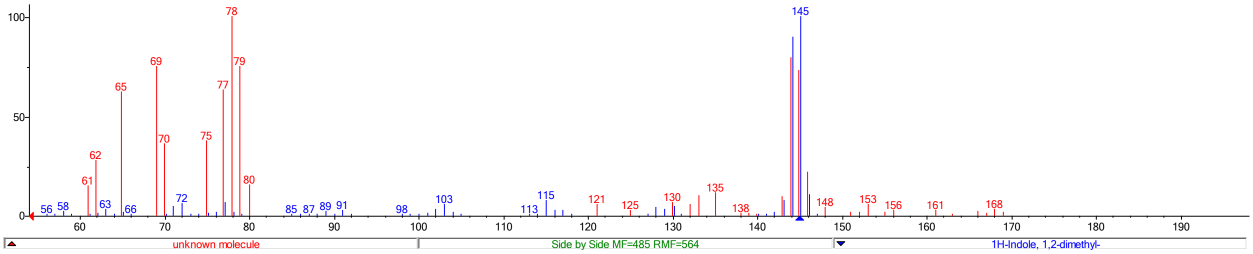

****

figure S-25. Gas chromatograph comparison between SAM-Flight model spectra and closest NIST match for Peak 23. (A) Gas Chromatograph 2 Peak 23 Retention time 14295 sec SAM-Flight Model spectra

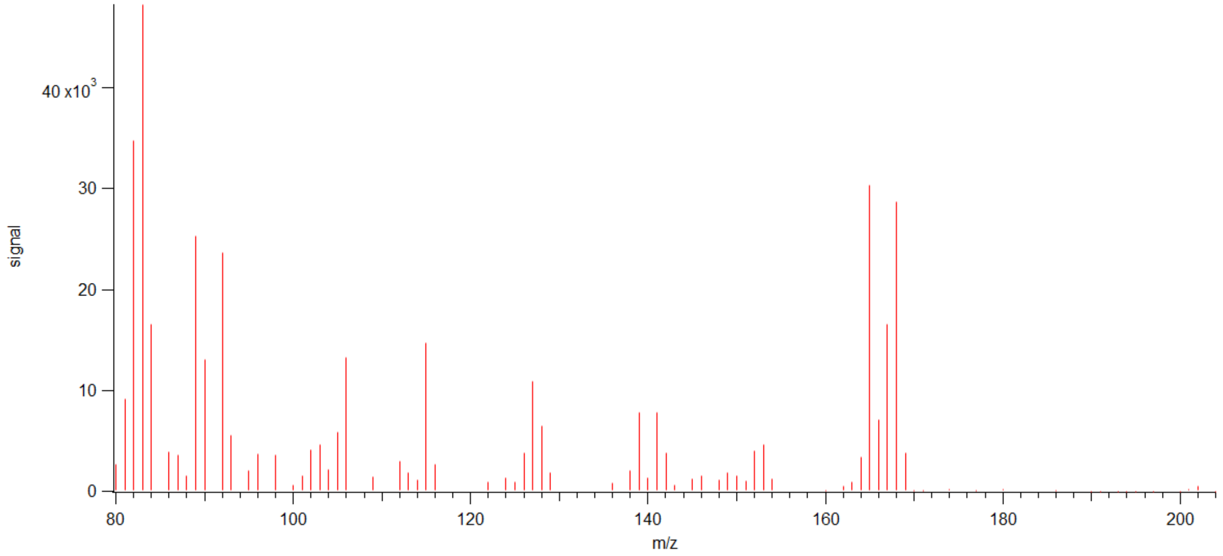

(B) Gas Chromatograph 2 Peak 23 SAM-Flight Model spectra closest NIST matches: Diphenylmethane AND/OR

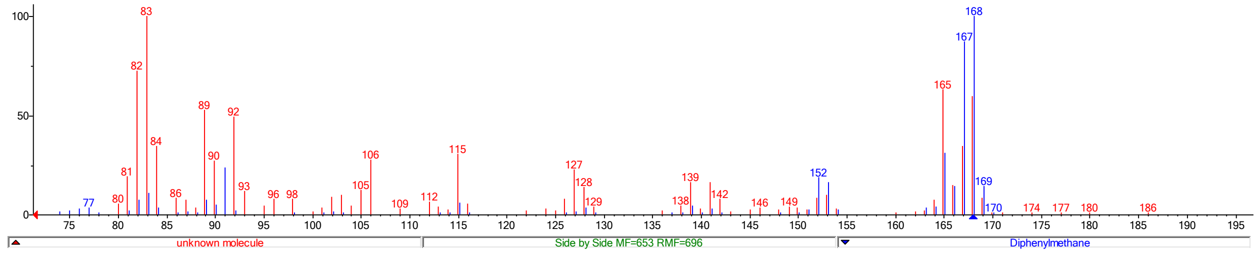

1,1-biphenyl, 2-methyl-

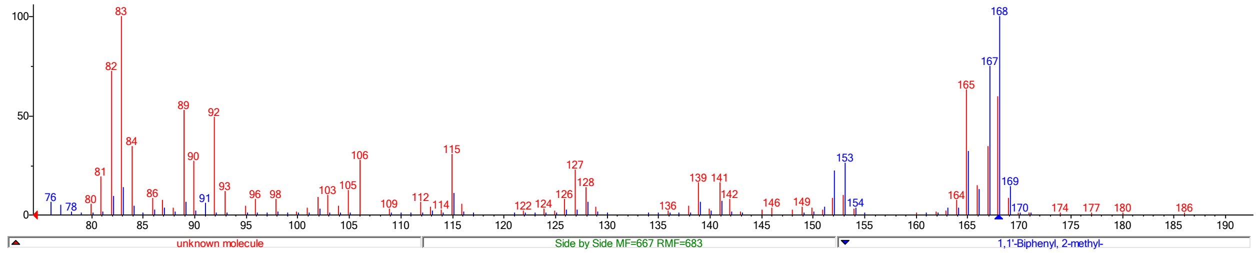

****

figure S-26. Gas chromatograph comparison between SAM-Flight model spectra and closest NIST match for Peak 24. (A) Gas Chromatograph 2 Peak 24 Retention time 14311 sec SAM-Flight Model spectra

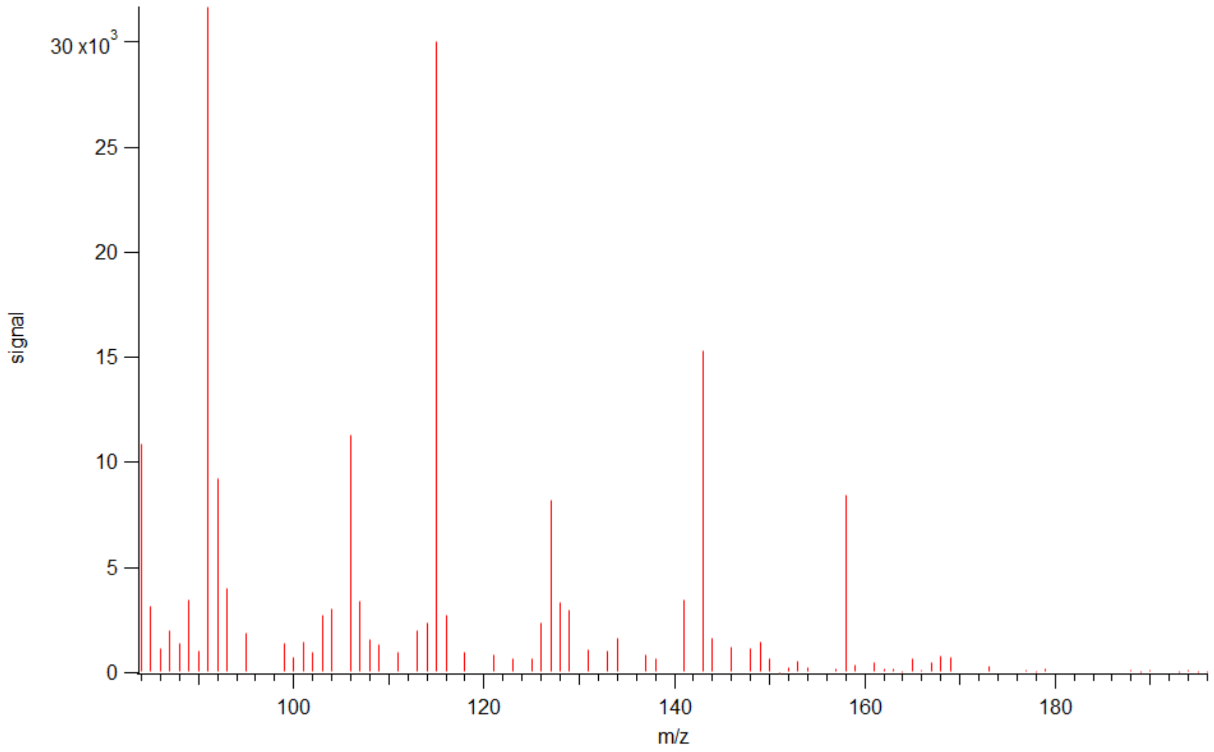

(B) Gas Chromatograph 2 Peak 24 SAM-Flight Model spectra closest NIST match: 1-Methoxynaphthalene

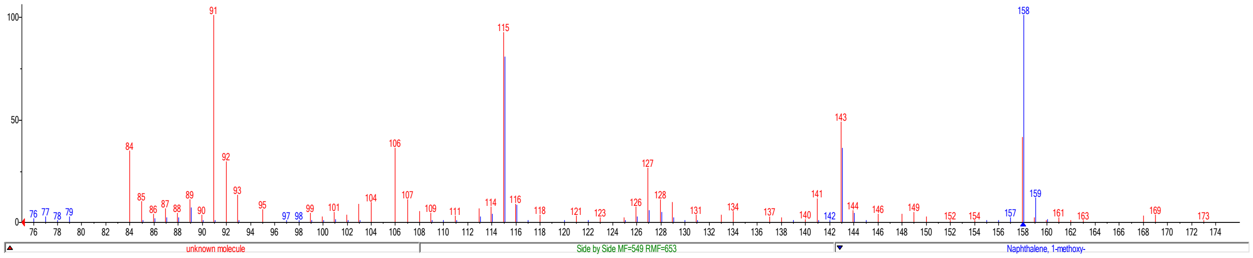

figure. S-27. Diagram detailing the SAM instrument suite, sample cups, and relevant chemical reactions. (A) SAM instrument suite with side panels removed. (B) Examples of the foil-capped metal cups for wet chemistry experiments and the quartz cups for evolved gas and standard mass spectrometry analysis of solid samples. (C) A foil-capped wet chemistry cup (image of foil cap in inset). (D) Interior schematic of wet chemistry cup. (E) Derivatization and thermochemolysis reactions with a) MTBSTFA/ DMF and b) TMAH/ MeOH.

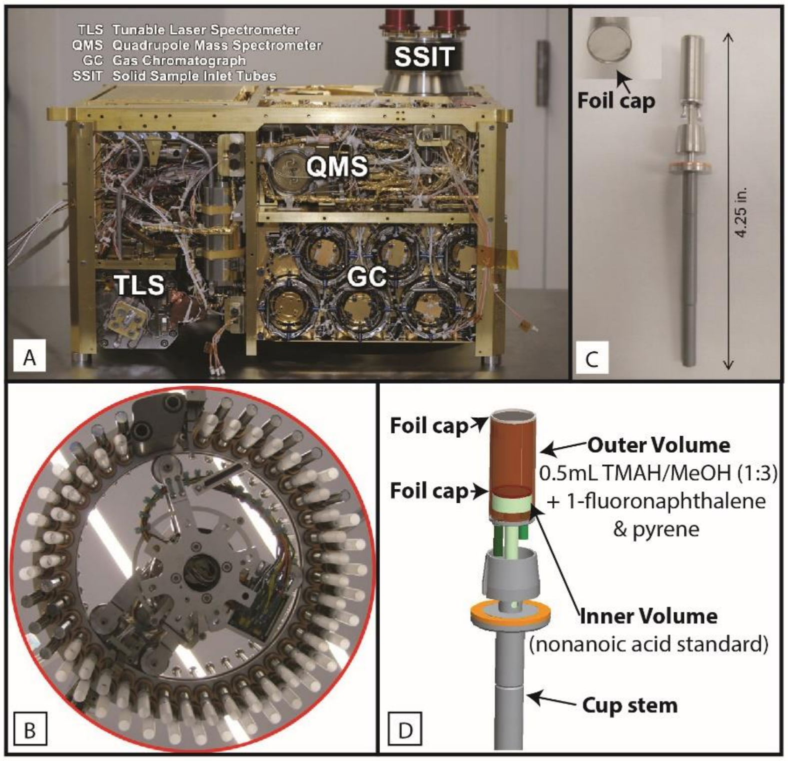

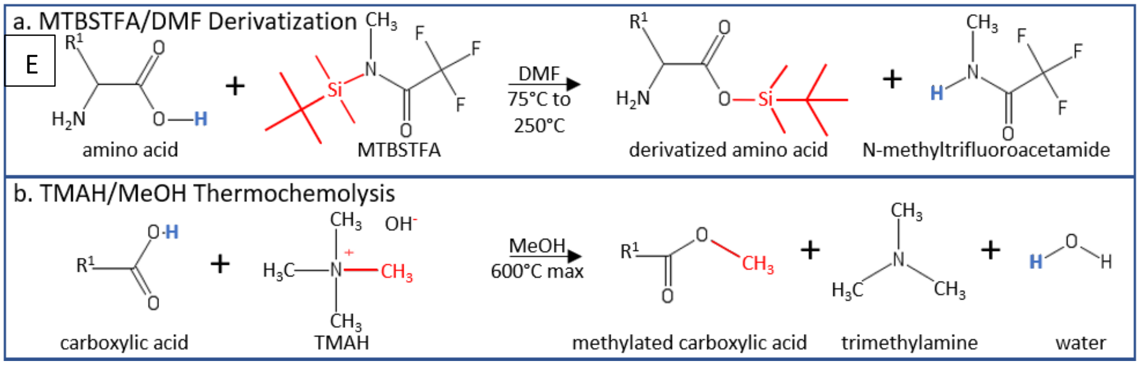

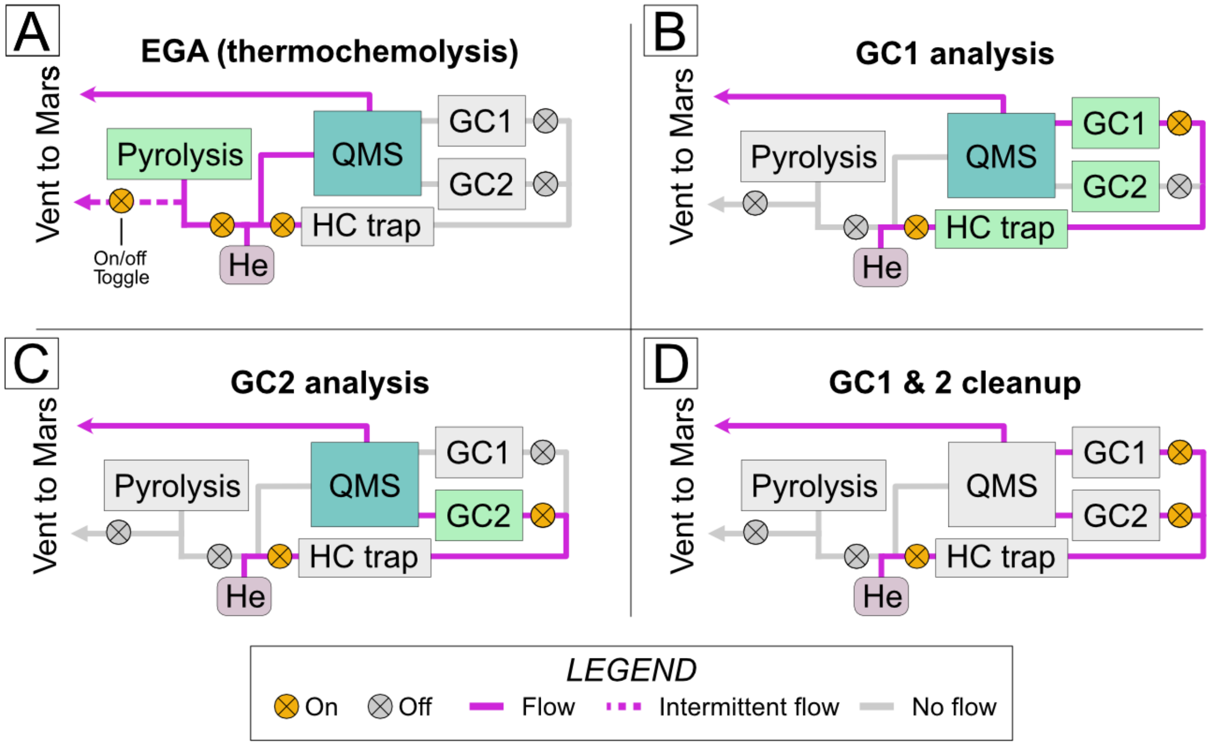

figure S-28. Simplified flow pathways for different configurations of the Sample Analysis at Mars (SAM) instrument. In each diagram, light green boxes represent the location where sample gases are either being generated or trapped. The corresponding blue boxes show where that sample gas is being analyzed. A) During evolved gas analysis (EGA), sample material is heated in the pyrolysis oven, and the resulting gases are transported via He flow to the QMS for analysis. Crucially, there is an on/off toggle that enables the instrument to "sniff" the sample without exceeding the detector capabilities. B) During a Gas Chromatograph 1 analysis, sample gas flows from the hydrocarbon (HC) trap through Gas Chromatograph 1 to the QMS and is simultaneously adsorbed onto Gas Chromatograph 2. C) At the start of a Gas Chromatograph 2 analysis, sample previously adsorbed to Gas Chromatograph 2 during a Gas Chromatograph 1 analysis is pumped into the QMS for analysis. D) Gas Chromatograph cleanup procedures simultaneously function as a method of cleaning the hydrocarbon trap, Gas Chromatograph 1, and Gas Chromatograph 2 as well as monitoring contamination in the instrument. Material released during a Gas Chromatograph cleanup can be analyzed on the quadrupole mass spectrometer just like during a Gas Chromatograph 1 or Gas Chromatograph 2 analysis.

Supplementary References

1. A. Mojarro, A. Buch, J. P. Dworkin, J. L. Eigenbrode, C. Fressinet, D. P. Glavin, C. Szopa, M. Millan, A. J. Williams, R. E. Summons, Murchison Meteorite Analysis Using Tetramethylammonium Hydroxide (TMAH) Thermochemolysis Under Simulated Sample Analysis at Mars (SAM) Pyrolysis-Gas Chromatography-Mass Spectrometry Conditions. *J. Geophys. Res. Planets* **128**, e2023JE007968 (2023).

2. A. Buch, I. Belmahdi, C. Szopa, C. Freissinet, D. P. Glavin, M. Millan, R. Summons, D. Coscia, S. Teinturier, J. Y. Bonnet, Y. He, M. Cabane, R. Navarro-Gonzalez, C. A. Malespin, J. Stern, J. Eigenbrode, P. R. Mahaffy, S. S. Johnson, Role of the Tenax® Adsorbent in the Interpretation of the EGA and GC-MS Analyses Performed With the Sample Analysis at Mars in Gale Crater. *J. Geophys. Res. Planets* **124**, 2819–2851 (2019).

3. K. E. Miller, B. Kotrc, R. E. Summons, I. Belmahdi, A. Buch, J. L. Eigenbrode, C. Freissinet, D. P. Glavin, C. Szopa, Evaluation of the Tenax trap in the Sample Analysis at Mars instrument suite on the Curiosity rover as a potential hydrocarbon source for chlorinated organics detected in Gale Crater. *J. Geophys. Res. Planets* **120**, 1446–1459 (2015).

4. M. Millan, S. Teinturier, C. Malespin, J.-Y. Bonnet, A. Buch, J. Dworkin, J. Eigenbrode, C. Freissinet, D. Glavin, R. Navarro-Gonzalez, A. Srivastava, J. Stern, B. Sutter, C. Szopa, A. J. Williams, R. Williams, G. Wong, P. Mahaffy, S. Johnson, Detection of Organic Molecules on Mars using the Curiosity Rover's Derivatization Experiment. *Nat. Astron.* **6**, 129–140 (2022).

Figure Legends

figure S-1. Gas chromatograph comparison between SAM-Flight model spectra and closest NIST match for Peak 1. (A) Gas Chromatograph 1 Peak 1 Retention time 10088sec SAM-Flight Model spectra (B) Gas Chromatograph 1 Peak 1 SAM-Flight Model spectra closest NIST match: 1,3,5-Trimethylbenzene.

figure S-2. Gas chromatograph comparison between SAM-Flight model spectra and closest NIST match for Peak 2. (A) Gas Chromatograph 1 Peak 2 Retention time 10196 sec SAM-Flight Model spectra (B) Gas Chromatograph 1 Peak 2 SAM-Flight Model spectra closest NIST match: 1,2,3,5-Tetramethylbenzene.

figure S-3. Gas chromatograph comparison between SAM-Flight model spectra and closest NIST match for Peak 3. (A) Gas Chromatograph 1 Peak 3 Retention time 10239 sec SAM-Flight Model spectra (B) Gas Chromatograph 1 Peak 3 SAM-Flight Model spectra closest NIST matches: Benzenemethanol, 4-(1-methylethyl)- AND/OR Retention time 10239 Trimethyl-1,4-benzenediamine.

figure S-4. Gas chromatograph comparison between SAM-Flight model spectra and closest NIST match for Peak 4. (A) Gas Chromatograph 1 Peak 4 Retention time 10278 sec SAM-Flight Model spectra (B) Gas Chromatograph 1 Peak 4 SAM-Flight Model spectra closest NIST matches: Retention time 10278 Trimethyl-1,4-benzenediamine AND/OR Retention time 10278 ethyltetramethylcyclopentadiene AND/OR Retention time 10278 benzoic acid 2 methyl methyl ester.

figure S-5. Gas chromatograph comparison between SAM-Flight model spectra and closest NIST match for Peak 6. (A) Gas Chromatograph 1 Peak 6 Retention time 10316 sec SAM-Flight Model spectra of Recovery Standard 1-fluoronaphthalene.

figure S-6. Gas chromatograph comparison between SAM-Flight model spectra and closest NIST match for Peak 7. (A) Gas Chromatograph 1 Peak 7 Retention time 10330 sec SAM-Flight Model spectra (overlap with *m/z* 146 of 1-fluoronaphthalene from Retention time10316 remains) (B) Gas Chromatograph 1 Peak 7 SAM-Flight Model spectra closest NIST match: Naphthalene.

figure S-7. Gas chromatograph comparison between SAM-Flight model spectra and closest NIST match for Peak 8. (A) Gas Chromatograph 1 Peak 8 Retention time 10355 sec SAM-Flight

Model spectra (B) Gas Chromatograph 1 Peak 8 SAM-Flight Model spectra closest NIST match: Benzothiophene.

figure S-8. Gas chromatograph comparison between SAM-Flight model spectra and closest NIST match for Peak 9. (A) Gas Chromatograph 2 Peak 9 Retention time 13683 sec SAM-Flight Model spectra (B) Gas Chromatograph 2 Peak 9 SAM-Flight Model spectra closest NIST match: Benzenamine, N,N,2-trimethyl-.

figure S-9. Gas chromatograph comparison between SAM-Flight model spectra and closest NIST match for Peak 10. (A) Gas Chromatograph 2 Peak 10 Retention time 13767 sec SAM-Flight Model spectra (B) Gas Chromatograph 2 Peak 10 SAM-Flight Model spectra closest NIST match: Benzenamine, N,N-dimethyl-.

figure S-10. Gas chromatograph comparison between SAM-Flight model spectra and closest NIST match for Peak 11. (A) Gas Chromatograph 2 Peak 11 Retention time 13790 sec SAM-Flight Model spectra (B) Gas Chromatograph 2 Peak 11 SAM-Flight Model spectra closest NIST match: Benzoic acid methyl ester.

figure S-11. Gas chromatograph comparison between SAM-Flight model spectra and closest NIST match for Peak 12. (A) Gas Chromatograph 2 Peak 12 Retention time 13837 sec SAM-Flight Model spectra (B) Gas Chromatograph 2 Peak 12 SAM-Flight Model spectra closest NIST matches: 1,4-Dimethylanisole AND/OR 3, 4, 5-trimethylphenol.

figure S-12. Gas chromatograph comparison between SAM-Flight model spectra and closest NIST match for Peak 13. (A) Gas Chromatograph 2 Peak 13 Retention time 13864 sec SAM-Flight Model spectra (B) Gas Chromatograph 2 Peak 13 SAM-Flight Model spectra closest NIST matches: Benzene, 1,2,4,5-tetramethyl- AND/OR o-Cymene AND/OR Benzene, 2-ethyl, 1,4-dimethyl.

figure S-13. Gas chromatograph comparison between SAM-Flight model spectra and closest NIST match for Peak 14. (A) Gas Chromatograph 2 Peak 14 Retention time 13942 sec SAM-Flight Model spectra (B) Gas Chromatograph 2 Peak 14 SAM-Flight Model spectra closest NIST matches: Benzene, 1,4-diethyl- AND/OR Benzene, 2-ethyl-1,4-dimethyl- AND/OR Benzene, 4-ethyl-1,2-dimethyl-.

figure S-14. Gas chromatograph comparison between SAM-Flight model spectra and closest NIST match for Peak 15. (A) Gas Chromatograph 2 Peak 15 Retention time 13948 sec SAM-Flight Model spectra (B) Gas Chromatograph 2 Peak 15 SAM-Flight Model spectra closest NIST match: Naphthalene, 1,2-dihydro-.

figure S-15. Gas chromatograph comparison between SAM-Flight model spectra and closest NIST match for Peak 16. (A) Gas Chromatograph 2 Peak 16 Retention time 13965 sec SAM-Flight Model spectra (B) Gas Chromatograph 2 Peak 16 SAM-Flight Model spectra closest NIST matches: benzene 1-methoxy-4-(1-methylethyl) AND/OR phenol, 2, 3, 5, 6-tetramethyl-.

figure S-16. Gas chromatograph comparison between SAM-Flight model spectra and closest NIST match for Peak 17. (A) Gas Chromatograph 2 Peak 17 Retention time 13984 sec SAM-

Flight Model spectra (B) Gas Chromatograph 2 Peak 17 SAM-Flight Model spectra closest NIST match: NN2,4-tetramethylbenzenamine.

figure S-17. Gas chromatograph comparison between SAM-Flight model spectra and closest NIST match for Peak 6. (A) Gas Chromatograph 2 Peak 6 Retention time 14007 sec SAM-Flight Model spectra of Recovery Standard 1-fluoronaphthalene (B) Gas Chromatograph 2 Peak 6 SAM-Flight Model spectra closest NIST match: 1-fluoronaphthalene (Recovery Standard).

figure S-18. Gas chromatograph comparison between SAM-Flight model spectra and closest NIST match for Peak 7. (A) Gas Chromatograph 2 Peak 7 Retention time 14012 sec SAM-Flight Model spectra (B) Gas Chromatograph 2 Peak 7 SAM-Flight Model spectra closest NIST matches: Naphthalene.

figure S-19. Gas chromatograph comparison between SAM-Flight model spectra and closest NIST match for Peak 8. (A) Gas Chromatograph 2 Peak 8 Retention time 14021 sec SAM-Flight Model spectra (B) Gas Chromatograph 2 Peak 8 SAM-Flight Model spectra closest NIST matches: Benzothiophene.

figure S-20. Gas chromatograph comparison between SAM-Flight model spectra and closest NIST match for Peak 18. (A) Gas Chromatograph 2 Peak 18 Retention time 14078 sec SAM-Flight Model spectra (B) Gas Chromatograph 2 Peak 18 SAM-Flight Model spectra closest NIST matches: phenol, 2, 3, 5, 6-tetramethyl- AND/OR NNN-trimethyl-1,4-benzenediamine.

figure S-21. Gas chromatograph comparison between SAM-Flight model spectra and closest NIST match for Peak 19. (A) Gas Chromatograph 2 Peak 19 Retention time 14125 sec SAM-Flight Model spectra (B) Gas Chromatograph 2 Peak 19 SAM-Flight Model spectra closest NIST match: naphthalene, 1,2-dihydro-3-methyl.

figure S-22. Gas chromatograph comparison between SAM-Flight model spectra and closest NIST match for Peak 20. (A) Gas Chromatograph 2 Peak 20 Retention time 14143 sec SAM-Flight Model spectra (B) Gas Chromatograph 2 Peak 20 SAM-Flight Model spectra closest NIST match: Benzene, pentamethyl.

figure S-23. Gas chromatograph comparison between SAM-Flight model spectra and closest NIST match for Peak 21. (A) Gas Chromatograph 2 Peak 21 Retention time 14163 sec SAM-Flight Model spectra (B) Gas Chromatograph 2 Peak 21 SAM-Flight Model spectra closest NIST match: Retention time 14163 2-Methylnaphthalene OR Retention time 14163 1-Methylnaphthalene.

figure S-24. Gas chromatograph comparison between SAM-Flight model spectra and closest NIST match for Peak 22. (A) Gas Chromatograph 2 Peak 22 Retention time 14236 sec SAM-Flight Model spectra (B) Gas Chromatograph 2 Peak 22 SAM-Flight Model spectra closest NIST match: Dimethyl-indole.

figure S-25. Gas chromatograph comparison between SAM-Flight model spectra and closest NIST match for Peak 23. (A) Gas Chromatograph 2 Peak 23 Retention time 14295 sec SAM-

Flight Model spectra (B) Gas Chromatograph 2 Peak 23 SAM-Flight Model spectra closest NIST matches: diphenylmethane AND/OR 1,1-biphenyl, 2-methyl-.

figure S-26. Gas chromatograph comparison between SAM-Flight model spectra and closest NIST match for Peak 24. (A) Gas Chromatograph 2 Peak 24 Retention time 14311 sec SAM-Flight Model spectra (B) Gas Chromatograph 2 Peak 24 SAM-Flight Model spectra closest NIST match: 1-Methoxynaphthalene.

figure. S-27. Diagram detailing the SAM instrument suite, sample cups, and relevant chemical reactions. (A) SAM instrument suite with side panels removed. (B) Examples of the foil-capped metal cups for wet chemistry experiments and the quartz cups for evolved gas and standard mass spectrometry analysis of solid samples. (C) A foil-capped wet chemistry cup (image of foil cap in inset). (D) Interior schematic of wet chemistry cup. (E) Derivatization and thermochemolysis reactions with a) MTBSTFA/ DMF and b) TMAH/ MeOH.

figure S-28. Simplified flow pathways for different configurations of the Sample Analysis at Mars (SAM) instrument. In each diagram, light green boxes represent the location where sample gases are either being generated or trapped. The corresponding blue boxes show where that sample gas is being analyzed. A) During evolved gas analysis (EGA), sample material is heated in the pyrolysis oven, and the resulting gases are transported via He flow to the QMS for analysis. Crucially, there is an on/off toggle that enables the instrument to "sniff" the sample without exceeding the detector capabilities. B) During a Gas Chromatograph 1 analysis, sample gas flows from the hydrocarbon (HC) trap through Gas Chromatograph 1 to the QMS and is simultaneously adsorbed onto Gas Chromatograph 2. C) At the start of a Gas Chromatograph 2 analysis, sample previously adsorbed to Gas Chromatograph 2 during a Gas Chromatograph 1 analysis is pumped into the QMS for analysis. D) Gas Chromatograph cleanup procedures simultaneously function as a method of cleaning the hydrocarbon trap, Gas Chromatograph 1, and Gas Chromatograph 2 as well as monitoring contamination in the instrument. Material released during a Gas Chromatograph cleanup can be analyzed on the quadrupole mass spectrometer just like during a Gas Chromatograph 1 or Gas Chromatograph 2 analysis.

Table Legends

table S-3. List of confirmed and potential molecule identities from SAM-Flight Model TMAH experiment and analytical standards analyzed with the SAM-Breadboard and flight spare columns. Not every potential identity was compared to an analytical standard. The retention time (Rt) comparison between the molecule on SAM-Flight Model and the SAM-Breadboard is reported for those standards that were analyzed. Noted as Y for yes are those molecules that were also detected in the Murchison meteorite with SAM-like neat pyrolysis or TMAH (tetramethylammonium hydroxide) thermochemolysis from Mojarro et al. (2023). * Denotes known SAM byproducts or background.

table S-4. Identification of molecules in evolved gas analysis (EGA), Gas Chromatograph 1, and Gas Chromatograph 2, with temperature range of evolution (EGA only), complementary peak number on chromatograms in Figs. 1 and 2, molecular ion, three highest abundance masses for each molecule, SAM experiment retention time, SAM retention time scaled to each channel, benchtop gas chromatograph mass spectrometry (GC-MS) retention time for comparison with candidate molecules, and difference in retention time between the SAM and benchtop comparisons, where applicable. * Denotes known SAM byproducts or background.
